# Blended Self-Management Interventions to Reduce Disease Burden in Patients With Chronic Obstructive Pulmonary Disease and Asthma: Systematic Review and Meta-analysis

**DOI:** 10.2196/24602

**Published:** 2021-03-31

**Authors:** Xiaoyue Song, Cynthia Hallensleben, Weihong Zhang, Zongliang Jiang, Hongxia Shen, Robbert J J Gobbens, Rianne M J J Van Der Kleij, Niels H Chavannes, Anke Versluis

**Affiliations:** 1 Department of Public Health and Primary Care Leiden University Medical Center Leiden Netherlands; 2 Faculty of Nursing and Health Zhengzhou University Zhengzhou China; 3 Faculty of Health, Sports and Social Work Inholland University of Applied Sciences Amsterdam Netherlands; 4 Zonnehuisgroep Amstelland Amstelveen Netherlands; 5 Department Family Medicine and Population Health Faculty of Medicine and Health Sciences University of Antwerp Antwerp Belgium

**Keywords:** blended intervention, COPD, asthma, meta-analysis, systematic review

## Abstract

**Background:**

Chronic obstructive pulmonary disease (COPD) and asthma have a high prevalence and disease burden. Blended self-management interventions, which combine eHealth with face-to-face interventions, can help reduce the disease burden.

**Objective:**

This systematic review and meta-analysis aims to examine the effectiveness of blended self-management interventions on health-related effectiveness and process outcomes for people with COPD or asthma.

**Methods:**

PubMed, Web of Science, COCHRANE Library, Emcare, and Embase were searched in December 2018 and updated in November 2020. Study quality was assessed using the Cochrane risk of bias (ROB) 2 tool and the Grading of Recommendations, Assessment, Development, and Evaluation.

**Results:**

A total of 15 COPD and 7 asthma randomized controlled trials were included in this study. The meta-analysis of COPD studies found that the blended intervention showed a small improvement in exercise capacity (standardized mean difference [SMD] 0.48; 95% CI 0.10-0.85) and a significant improvement in the quality of life (QoL; SMD 0.81; 95% CI 0.11-1.51). Blended intervention also reduced the admission rate (relative ratio [RR] 0.61; 95% CI 0.38-0.97). In the COPD systematic review, regarding the exacerbation frequency, both studies found that the intervention reduced exacerbation frequency (RR 0.38; 95% CI 0.26-0.56). A large effect was found on BMI (*d*=0.81; 95% CI 0.25-1.34); however, the effect was inconclusive because only 1 study was included. Regarding medication adherence, 2 of 3 studies found a moderate effect (*d*=0.73; 95% CI 0.50-0.96), and 1 study reported a mixed effect. Regarding self-management ability, 1 study reported a large effect (*d*=1.15; 95% CI 0.66-1.62), and no effect was reported in that study. No effect was found on other process outcomes. The meta-analysis of asthma studies found that blended intervention had a small improvement in lung function (SMD 0.40; 95% CI 0.18-0.62) and QoL (SMD 0.36; 95% CI 0.21-0.50) and a moderate improvement in asthma control (SMD 0.67; 95% CI 0.40-0.93). A large effect was found on BMI (*d*=1.42; 95% CI 0.28-2.42) and exercise capacity (*d*=1.50; 95% CI 0.35-2.50); however, 1 study was included per outcome. There was no effect on other outcomes. Furthermore, the majority of the 22 studies showed some concerns about the ROB, and the quality of evidence varied.

**Conclusions:**

In patients with COPD, the blended self-management interventions had mixed effects on health-related outcomes, with the strongest evidence found for exercise capacity, QoL, and admission rate. Furthermore, the review suggested that the interventions resulted in small effects on lung function and QoL and a moderate effect on asthma control in patients with asthma. There is some evidence for the effectiveness of blended self-management interventions for patients with COPD and asthma; however, more research is needed.

**Trial Registration:**

PROSPERO International Prospective Register of Systematic Reviews CRD42019119894; https://www.crd.york.ac.uk/prospero/display_record.php?RecordID=119894

## Introduction

### Background

Chronic lung diseases are the leading cause of disability and death worldwide [1]. Of all chronic lung diseases, chronic obstructive pulmonary disease (COPD) and asthma are the most prevalent [1]. There were approximately 251 million cases of COPD globally in 2015, and COPD is predicted to become the third leading cause of death by 2030 [2]. Approximately 300 million people have asthma worldwide, with a projected increase of an additional 100 million people by 2025 [3]. The impact of a health problem, measured by financial cost, morbidity, and other indicators, is called disease burden. It is often quantified in terms of disability-adjusted life years (DALYs) or quality-adjusted life years (QALYs) [1]. In 2017, the loss of DALYs was the first for COPD and the second for asthma [1]. In addition, a loss in health-related quality of life (QoL) is seen in many patients (eg, a decline in health, increased hospital admissions, and high medication costs). The World Health Organization estimates that the cost of a QALY for COPD ranges from US $6700 to $13,400 due to exacerbations and medication. In patients with asthma, annual costs vary from less than US $150 to US $3000 [4,5]. There is increased awareness that self-management represents a promising strategy to decrease disease burden [6]. Self-management could improve patient outcomes and decrease disease burden by supporting patients to positively adapt their health behaviors and develop skills to better manage their diseases [7].

Self-management refers to an individual’s ability to manage their symptoms, treatment, physical and psychosocial consequences, and lifestyle changes inherent to life with a chronic condition [8]. In traditional face-to-face self-management interventions, patients with COPD and asthma are equipped with the knowledge and skills to manage their health condition successfully [9]. Previous studies have found that these self-management interventions are effective in improving disease knowledge and self-efficacy [10]. However, these face-to-face self-management interventions are limited by their accessibility (eg, lower accessibility for patients who are more distant to the health care provider or when the health care provider lacks time) [11].

eHealth is an alternative to traditional face-to-face interventions. The most cited definition of eHealth is “health services and information delivered or enhanced through the internet and related technologies” [12]. Compared with traditional face-to-face interventions, eHealth interventions can be cost and time saving and offer better accessibility and flexibility [13]. Moreover, eHealth interventions can help optimize the therapeutic process, increase treatment efficiency, and decrease costs by enhancing (web-based) communication possibilities between health care providers and patients [14]. There have been promising results with eHealth self-management interventions [15,16]. A meta-analysis showed that, for patients with COPD, eHealth self-management programs (eg, web-based phone calls and web-based interventions) led to a significant improvement in symptoms [15]. However, eHealth interventions typically allow for limited tailoring of patients’ needs and lower patient engagement [17]. There have also been concerns about reliability, security, confidentiality, and lack of education and training [18]. These factors can negatively impact the implementation and effectiveness of these interventions.

The most recent development is the blended intervention. There are different definitions of blended interventions [19,20]. We use the definition by Erbe et al [20]: “Treatment programs that use elements of both face-to-face and internet-based interventions, including both the integrated and the sequential use of both treatment formats.” Blended interventions could retain the positive aspects of face-to-face interventions and eHealth by mitigating their negative aspects. Furthermore, blended intervention could diminish the number of face-to-face contacts needed and provide support that is available at all times [21]. With eHealth, patients can also monitor their health condition throughout the day and convey their health information to health care providers without time and distance limitations. Patients can also receive quick assistance during critical periods of care facilitated by real-time alerts and reminders, which could help patients adhere to their action plan. For patients with COPD and asthma, blended interventions can include various elements [22,23] (eg, training, education, and action plans) with different blended intervention components (eg, internet-based phone calls and individual face-to-face interventions, web platforms combined with individual face-to-face interventions) [22,23]. Some studies have shown that blended self-management interventions are effective in improving QoL in patients with COPD and asthma [24,25].

Current reviews suggest that blended interventions could be effective [19,20], but these reviews are limited for several reasons. First, the reviews focus on mental health and not on chronic lung diseases [20]. Second, the reviews focus on health-related effectiveness outcomes and not on process outcomes [19]. Third, the reviews do not specifically focus on self-management interventions [19,20]. To conclude, a comprehensive overview or meta-analysis of the effect of blended self-management interventions on the disease burden of patients with COPD and asthma, including process outcomes and health-related effectiveness outcomes, is lacking.

### Objectives

A systematic review will be performed to assess the effectiveness of blended self-management interventions in patients with COPD and asthma. When appropriate, a meta-analysis will be conducted. Internet-based, telephone, and SMS-delivered interventions are included because all of these are parts of eHealth [13]. Thus, this study aims to investigate the effectiveness of blended self-management interventions in patients with COPD and asthma.

## Methods

### Systematic Review and Meta-analysis

This review follows the PRISMA (Preferred Reporting Items for Systematic Reviews and Meta-Analyses) guidelines [26]. The review was registered in PROSPERO (number 2019: CRD42019119894).

### Search Strategy

A search strategy was established in collaboration with a certified librarian to identify relevant studies on blended self-management interventions in patients with COPD and asthma. A total of 5 electronic databases (ie, PubMed, Web of Science, COCHRANE Library, Emcare, and Embase) were searched on December 28, 2018, and updated on November 30, 2020. There were search terms related to 4 areas: (1) COPD or asthma, (2) eHealth, (3) face-to-face intervention, and (4) blended intervention (Multimedia Appendix 1). The search terms related to COPD or asthma and blended interventions were first combined, resulting in 84 studies. Due to the limited number of studies, the search terms associated with COPD or asthma were combined with terms about eHealth and face-to-face interventions. In every database, the search was limited to peer-reviewed publications. The search strategy was not restricted based on publication year, as we aimed to provide a comprehensive overview of the use of blended interventions in patients with COPD and asthma. In addition, reference lists of the included studies and previous reviews were searched to identify additional studies that might be eligible for inclusion.

### Eligibility Criteria

The patient, intervention, comparison, outcome, study design tool was used to develop an effective search strategy and determine the inclusion and exclusion criteria [27]. The following inclusion criteria were used to identify the studies: (1) participants: adults (≥18 years old) with COPD or asthma; (2) intervention: blended self-management intervention (consisting of an eHealth component combined with a face-to-face component); (3) comparison: eHealth intervention with or without usual care (UC) and face-to-face intervention with or without UC or only UC; (4) outcome measures: health-related effectiveness or process outcomes; and (5) individual randomized controlled trials (RCTs). Studies were excluded if: (1) the participants were children or adolescents, (2) the eHealth apps were only used to collect data, (3) outcomes were not about the health-related outcomes, and (4) studies were cluster RCTs.

### Study Selection

After the removal of duplicates, the identified titles and abstracts were screened for eligibility. If insufficient information was provided, the full-text paper was screened. When a full-text paper was not available, a request was sent to the authors. Studies that did not meet the inclusion criteria were excluded. Screening the titles, abstracts, and full texts was performed by 2 reviewers independently (XS and ZJ). Any disagreements between the 2 authors were resolved by a third reviewer (CH).

### Data Collection and Coding

Data were collected using a standardized data extraction form. It included (1) study characteristics (eg, first author, publication year, country, number and age of patients, percentage of female patients, disease severity or diagnosis, setting [ie, home, primary care (PC), or secondary care (SC)]), intervention, and follow-up duration), (2) intervention characteristics (ie, category and functionality of the eHealth and face-to-face component), (3) behavior change techniques (BCTs) used in the blended self-management intervention, and (4) the health-related effectiveness and process outcomes. Information was extracted from each publication by XS and ZJ. Inter-rater reliability, as assessed with Cohen κ, indicated strong agreement (κ=0.90) [28].

COPD severity was classified based on the Global Initiative for Chronic Obstructive Lung Disease (GOLD) criteria [29]. Patients were considered to have COPD when the ratio between forced expiratory volume in 1s (FEV1) and full forced vital capacity (FVC) was <0.70. The degree of obstruction was defined as follows: (1) GOLD I: FEV1 ≥80% predicted (mild), (2) GOLD II: 50%≤FEV1<80% predicted (moderate), (3) GOLD III: 30%≤FEV1<50% predicted (severe), and (4) GOLD IV: FEV1 <30% predicted (very severe). There is no standard classification of severity for patients with asthma.

As mentioned above, different intervention characteristics were extracted from the publications. First, the eHealth component of the intervention was categorized as a mobile app; eg, phone call or SMS), an internet-assisted intervention (eg, web page, chat room), or multiple component interventions with multiple eHealth technologies. Second, the function of the eHealth app was categorized into informing, instructing, displaying, guiding, reminding or alerts, and communicating (ie, between provider and patients) [30]. Third, face-to-face interventions were classified as individual (eg, home visits, PC or SC visits) or group-based interventions (eg, group pulmonary rehabilitation). Fourth, the function of the face-to-face intervention was classified as (1) education: introduction of disease-related information and how to use eHealth, (2) training: provide information about self-management, (3) consultation: discuss individual action plan, (4) assessment: test and assess the patient’s performance, or (5) monitoring: provide reminders to improve intervention adherence [31,32].

Outcome indicators were classified into health-related effectiveness outcome or process outcome indicators. Health-related effectiveness outcome indicators included outcomes related to disease status and health condition (ie, exercise capacity, dyspnea, lung function, QoL, admission, mortality, exacerbation frequency, and BMI). Process outcome indicators included intermediate outcomes during the implementation process (eg, visits, satisfaction, costs, smoking, self-management ability, physical activity, medication and therapy adherence, psychosocial, symptom management, nutrition, and alcohol). A positive effect was ascribed when there was a significant positive effect of the intervention on the outcome measure compared with the control group (CG). When the outcome measure did not significantly differ between the intervention group (IG) and CG, it was rated as *no effect*. A mixed effect was ascribed when multiple measures were used to measure a similar outcome, and the effect on the measures was in different directions (eg, in the study by Garcia [22], there was a significant positive effect on inhaler treatment adherence, whereas there was no effect on oral treatment adherence).

### Quality Assessment

Study quality was assessed using the Cochrane risk of bias (ROB) 2 tool [33]. The tool assessed 5 domains of potential bias: (1) randomization, (2) deviations from the intended interventions (effect of assignment to intervention), (3) missing outcome data, (4) measurement of the outcome, and (5) selection of the reported result. Each domain had a few signaling questions. On the basis of the authors’ (XS and ZJ) responses to the signaling questions, a judgment on the ROB (*low, some concerns,* or *high*) for each domain could be made to assess the bias that might confound the study findings [33]. The quality of the clinical evidence was critically appraised using the Grading of Recommendations, Assessment, Development, and Evaluation (GRADE) system [34], which evaluated the risk for bias, inconsistency, indirectness, and imprecision for each outcome. Four categories were used to define the quality of evidence: high quality of evidence (the true effect lies close to that of the effect estimate), moderate quality of evidence (the true effect is likely to be close to the effect estimate, but there is a possibility that it is substantially different), low quality of evidence (the true effect may be substantially different from the effect estimate), and very low quality of evidence (the true effect is likely to be substantially different from the effect estimate) [35]. The quality assessment was performed by XS and ZJ, and any disagreements were resolved through discussion. Inter-rater reliability, as assessed with Cohen κ [28], indicated that there was strong agreement between raters (κ=0.80).

### Data Analysis

When an outcome was assessed using different measurements in one study, data from the most specific disease-related questionnaire were used. For example, in the study by Garcia [22], QoL was measured using both the Saint-George’s Respiratory Questionnaire (SGRQ), a specific QoL questionnaire, and Euroqol, a generic health-related QoL questionnaire. SGRQ was selected and analyzed in the meta-analysis because it is the most specific disease-related questionnaire.

First, a systematic review was conducted to determine the results. For continuous data, Cohen *d* was recommended to calculate the effect size [36] (Cohen *d* >0.2=small effect, Cohen *d* >0.5=moderate effect, and Cohen *d* >0.8=large effect) [37]. For dichotomous data, the relative ratio (RR) was calculated to assess the effect size. An RR greater than 1 indicates an increased likelihood that the stated outcome is achieved in the IG. If the RR is less than 1, there is a decreased likelihood that the outcome is achieved in the IG. A ratio of 1 indicated no difference (ie, the outcome was just as likely to occur in the IG as it was in the CG) [38].

When 3 or more studies reported on the same outcome measure, this outcome was included in the meta-analysis [39]. For continuous data, the standardized mean difference (SMD) accounted for the same outcomes measured with different assessment tools (eg, QoL was assessed using the SGRQ, COPD assessment test [CAT], and chronic respiratory questionnaire [CRQ]). SMDs were used to standardize the results of the studies to a uniform scale before they could be combined in the quantitative synthesis. SMDs and associated 95% CIs were used to calculate the mean difference and SD difference between the IG and CG for each study. When the mean or SD was not mentioned, the author was contacted for missing information. Cohen *d* was used to interpret the data [37]. For dichotomous data, RR was calculated to assess the effect size [38]. Publication bias was tested if more than 10 studies report on the same outcome measure [40]. *P*<.05 was considered significant for the effect estimate.

A random-effect model was used because the variance of study populations and intervention designs was anticipated as heterogeneity across the included studies [41]. Heterogeneity was assessed using chi-square tests and *I^2^* statistics [42]. A *P* value of <.1 indicates statistically significant heterogeneity. The *I*^2^ statistic was used to quantify the size of the heterogeneity between studies: 25%, 50%, and 75% can be considered small, medium, and substantial heterogeneity, respectively [42]. Outliers were identified using the value of the standardized residual [43]. Studies whose standardized residual was equal to or larger than 1.96 were identified as an outlier and were excluded from the meta-analysis. No subgroup analysis was planned because of the limited number of studies. All analyses were performed using the Review Manager (RevMan version 5.4; The Cochrane Collaboration) and Stata version 14.0 (StataCorp) [44].

## Results

### Search Results

**Figure 1:**
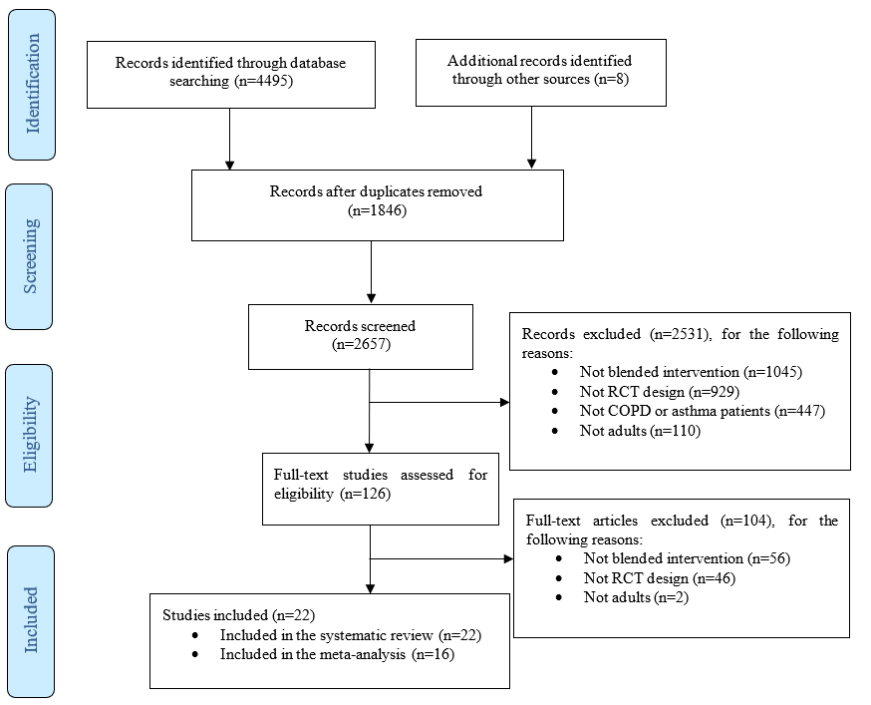
PRISMA (Preferred Reporting Items for Systematic Reviews and Meta-analyses) flowchart of the systematic review and meta-analysis. COPD: chronic obstructive pulmonary disease; RCT: randomized controlled trial.

The literature search identified a total of 4495 potentially eligible records, and 2657 records remained after duplicates were excluded. After screening the titles and abstracts, additional 2531 records were excluded for other reasons (Figure 1). The full texts of the remaining 126 studies were assessed, and 22 RCTs [22-25,45-62] were included in this review. Of the 22 RCTs, 2 were pilot RCT studies [45,51] and 1 was a feasibility RCT [48]. These studies were included because they followed the CONSORT (Consolidated Standards of Reporting Trials) checklist [45,51], and they were small sample size RCTs [48,51]. A total of 15 RCTs focused on patients with COPD [22,24,45-57]. Of these studies, 11 were included in the meta-analysis [22,24,45,47,48,50,51,54-57]. The remaining 4 studies [46,49,52,53] were excluded because no available means and SDs were reported or obtained after contacting the authors. A total of 7 studies focused on patients with asthma [23,25,58-62]. Of the 7 asthma studies with available data, 5 were pooled into a meta-analysis [25,58,60-62]. The other 2 studies were not included in the meta-analysis because of the lack of means and SDs after contacting the authors.

### Study Characteristics

A total of 15 COPD studies [22,24,45-57] were published between 2006 and 2020 and were conducted in China (n=5) [48,54-57], United States (n=2) [24,51], Denmark (n=2) [49,52], Canada (n=1) [53], England (n=1) [45], Spain (n=1) [22], Germany (n=1) [50], Australia (n=1) [46], and 1 in both Spain and Belgium [47]. The sample size ranged from 39 to 242 (with a total sample size of 1477). The average age of patients with COPD ranged from 64.10 to 73.50 years. Of the 15 COPD studies, 8 had UC as a CG [22,24,46,47,49,54,56,57], 5 had a *visit* as CG (meaning that the health care provider visited the patients’ home or patients visited the PC or SC) [45,48,51,52,54], and 2 studies had both UC and visits in the CG [50,53]. The settings were home and SC (n=9) [22,24,46,47,53-57], home care (n=2) [45,48], and home care and PC (n=4) [49-52]. The duration of the blended self-management interventions ranged from 4 to 48 weeks, with a mean of 22.13 weeks (SD 16.20). The follow-up duration ranged from 17 to 48 weeks.

Seven asthma studies [23,25,58-63] were published from 2003 to 2020 and were conducted in the Netherlands (n=3) [25,61,62], Germany (n=1) [59], England (n=1) [23], United States (n=1) [60], and China (n=1) [58]. The study sample size ranged from 16 to 200 (total N=527). The mean age of patients with asthma ranged from 24.80 to 52.00 years. CG included UC (n=4) [23,25,60,62] and visits (n=3) [58,59,61]. The duration of the blended self-management interventions ranged from 3 to 48 weeks, with a mean of 15.88 weeks (SD 13.48). The follow-up duration ranged from 36 to 120 weeks. An overview of the study characteristics is provided in Table 1.

**Table 1:** Study characteristics of chronic obstructive pulmonary disease and asthma studies.

COPD^a^ and asthma study^b^			Country		Participants				Setting		Participants, mean (SD)					Gender (female), n (%)			Severity^c^			CG^d^			Intervention (weeks)			Follow-up (weeks)	
					IG^e^		CG				IG		CG																
**COPD (included in the meta-analysis)**																													
	Bentley et al [45]	England		25		23		Home		67.20 (11.60)		65.90 (9.40)		—^f^			—			Home visits			8			32			
	Chau et al [48]	China		22		18		Home		73.50 (6.10)		72.20 (6.10)		1 (3)			II-IV			Home visits			8			—			
	Casas et al [47]	Spain and Belgium		65		90		Home and SC^g^		70.00 (90.00)		72.00 (90.00)		26 (16.8)			I-IV			UC^h^			4			48			
	Garcia et al [22]	Spain		21		41		Home and SC		73.00 (6.00)		74.00 (8.00)		8 (13)			—			UC			48			—			
	Jehn et al [50]	Germany		32		30		Home and PC^i^		64.10 (10.90)		69.10 (9.20)		14 (23)			II-IV			UC+PC visits			36			—			
	Koff et al [24]	United States		20		20		Home and SC		66.60 (9.10)		65.00 (8.20)		21 (53)			III-IV			UC			12			—			
	Nguyen et al [51]	United States		19		20		Home and PC		68.00 (8.30)		70.90 (8.60)		17 (44)			—			Home visits			24			—			
	Wang et al [54]	China		55		65		Home and SC		69.30 (7.80)		71.90 (8.10)		63 (52.5)			II-IV			UC			24			48			
	Wang et al [55]	China		39		39		Home and SC		63.20 (7.50)		64.40 (7.00)		23 (30)			Mostly II-IV			SC visits			48			—			
	Wei et al [56]	China		42		45		Home and SC		65.20 (8.10)		63.90 (6.20)		—			I-IV			UC			24			48			
	Xin et al [57]	China		114		113		Home and SC		64.20 (14.20)		64.60 (14.50)		141 (62.1)			—			UC			48			—			
**COPD (not included in the meta-analysis)**																													
	Cameron et al [46]	Australia		35		30		Home and SC		68.00 (9.90)		70.00 (6.80)		—			I-IV			UC			8			17			
	Haesum et al [49]	Denmark		47		43		Home and PC		70.20 (9.00)		69.50 (10.10)		47 (52)			I-IV			UC			4			40			
	Sorknaes et al [52]	Denmark		121		121		Home and PC		71.00 (10.00)		72.00 (9.00)		—			I-IV			PC visits			12			26			
	Stamenova et al [53]	Canada		41		41		Home and SC		71.98 (9.52)		71.76 (7.28)		36 (44)			II-IV			SC visits			24			—			
	Stamenova et al [53]	Canada		41		40		Home and SC		71.98 (9.52)		72.78 (9.16)		37 (46)			II-IV			UC			24			—			
**Asthma** **(included in the meta-analysis)**																													
	Cao et al [58]	China		37		30		Home and SC		39.10 (14.30)		41.40 (12.00)		52 (78)			—			SC visits			12			—			
	Ostojic et al [60]	United States		8		8		Home and PC		24.80 (6.30)		24.50 (7.00)		7 (44)			M^j^			UC			16			—			
	Türk et al [61]	The Netherlands		7		10		SC		41.57 (12.54)		41.90 (8.58)		13 (77)			—			SC visits			12			48			
	Türk et al [61]	The Netherlands		14		10		SC		41.57 (9.73)		41.90 (8.58)		19 (79)			—			SC visits			12			48			
	van der Meer et al [25]	The Netherlands		101		99		Home and SC		36.00 (19.00; 50.00)		37.00 (18.00; 50.00)		139 (69.5)			—			UC			12			36			
	van Gaalen et al [62]	The Netherlands		47		60		Home and SC		36.00 (8.70)		37.00 (8.00)		76 (71.0)			—			UC			48			120			
**Asthma (not included in the meta-analysis)**																													
	Barbanel et al [23]	England		12		12		Home and SC		45.00 (17.00)		47.00 (17.00)		13 (54)			—			UC			12			—			
	Kohler et al [59]	Germany		41		41		Home and PC		49.00 (12.00)		52.00 (8.00)		32 (39)			—			PC^i^ visits			3			—			

^a^COPD: chronic obstructive pulmonary disease.

^b^Study by Bentley et al [45] and Nguyen et al [51] were feasibility RCTs, and study by Chau et al [48] was a pilot RCT. There was 1 study including 1 intervention group and 2 control groups (study by Stamenova et al [53]). Study by Türk et al [61] included 2 intervention groups and 1 control group.

^c^COPD severity was classified according to GOLD (Global Initiative for Chronic Obstructive Lung Disease) classification. Asthma severity was classified by the physician diagnosis.

^d^CG: control group.

^e^IG: intervention group.

^f^Not reported in the study.

^g^SC: secondary care.

^h^UC: usual care.

^i^PC: primary care.

^j^M: moderate severity.

### Quality Assessment

#### Methodological Quality

The ROB is summarized in Table 2. Among the 15 COPD studies, the overall ROB was rated as *some concerns* in 10 studies [22,46,47,49,52-57] and *high* in 5 studies [24,45,48,50,51]. In addition, 2 studies had some concerns in the randomization process [48,50], and 13 studies showed a low ROB in the randomization process [22,24,45-47,49,51-57]. The majority of the studies showed some concerns [22,45-47,49,51-57], whereas 3 studies showed high risk from intended intervention [24,48,50]. A low ROB due to missing outcome data was found in 14 studies [22,24,45,46,48-57], whereas 1 study showed some concerns [47]. The ROB in the measurement of the outcome had some concerns in 13 studies [22,24,45,46,48-51,53-57] and a low ROB in 2 studies [47,52]. A low ROB in the selection of the reported result was found in the majority of studies [22,24,46-50,52-57], and 2 studies had some concerns [45,51].

In asthma studies, the overall ROB indicated some concerns in 4 studies [23,25,61,62] and high risk in 3 studies [58-60]. Four studies showed a low ROB in the randomization process [23,25,61,62], and 3 studies showed some concerns [58-60]. All studies indicated some concerns due to deviations from the intended intervention [23,25,58-62]. In total, 6 studies showed a low ROB outcome data [23,25,59-62], and 1 study had some concerns due to missing outcome data [58]. All studies showed some concerns in the measurement of the outcomes and low ROB in the selection of the reported results [23,25,58-62].

**Table 2:** Risk of bias judgments for chronic obstructive pulmonary disease and asthma randomized controlled trials.

COPD^a^ or asthma study			Bias arising from the randomization process		Bias due to deviations from the intended intervention		Bias due to missing outcome data		Bias in measurement of the outcome		Bias in selection of the reported result		Overall bias
**COPD**													
	Bentley et al [45]	L^b^		S^c^		L		S		S		H^d^	
	Cameron et al [46]	L		S		L		S		L		S	
	Casas et al [47]	L		S		S		L		L		S	
	Chau et al [48]	S		H		L		S		L		H	
	Garcia [22]	L		S		L		S		L		S	
	Haesum et al [49]	L		S		L		S		L		S	
	Jehn et al [50]	S		H		L		S		L		H	
	Koff et al [24]	L		H		L		S		L		H	
	Nguyen et al [51]	L		S		L		S		S		H	
	Sorknaes et al [52]	L		S		L		L		L		S	
	Stamenova et al [53]	L		S		L		S		L		S	
	Wang et al [54]	L		S		L		S		L		S	
	Wang et al [55]	L		S		L		S		L		S	
	Wei et al [56]	L		S		L		S		L		S	
	Xin et al [57]	L		S		L		S		L		S	
**Asthma**													
	Barbanel et al [23]	L		S		L		S		L		S	
	Cao et al [58]	S		S		S		S		L		H	
	Kohler et al [59]	S		S		L		S		L		H	
	Ostojic et al [60]	S		S		L		S		L		H	
	Türk et al [61]	L		S		L		S		L		S	
	van der Meer et al [25]	L		S		L		S		L		S	
	van Gaalen et al [62]	L		S		L		S		L		S	

^a^COPD: chronic obstructive pulmonary disease.

^b^L: low risk of bias.

^c^S: some concerns.

^d^H: high risk of bias.

#### Quality of Evidence

In COPD studies, 19 different outcome measures were included (ie, exercise capacity, dyspnea, lung function, QoL, admission rate, exacerbation frequency, mortality, BMI, visits, satisfaction, costs, smoking, medication adherence, self-management ability, physical activity, psychosocial, symptom management, nutrition, and alcohol). Two outcome measures were rated as high quality of evidence (ie, exercise capacity and mortality), 1 measure had a moderate quality of evidence (ie, admission rate), 6 had a low quality of evidence (ie, dyspnea, lung function, QoL, visits, satisfaction, and physical activity), and the other 10 showed very low quality of evidence (exacerbation frequency, BMI, adherence, self-management ability, smoking, costs, psychosocial, symptom management, nutrition, and alcohol). In asthma studies, 10 different outcome measures were included (ie, admission rate, BMI, exercise capacity, asthma control, lung function, QoL, asthma knowledge, adherence, visits, and exacerbation frequency). Of the 10 outcomes, 7 were rated as having very low quality of evidence (ie, admission rate, BMI, exercise capacity, asthma knowledge, adherence, visits, and exacerbation frequency). Asthma control, lung function, and QoL were rated as having moderate quality of evidence (Multimedia Appendix 2 [22-25,45-62]).

### Intervention Characteristic

#### Category of the Blended Self-Management Intervention

In COPD studies, 5 blended self-management intervention combinations were discussed: (1) multiple component eHealth and an individual face-to-face intervention (n=6) [49-53,57], (2) internet-assisted intervention and an individual face-to-face intervention (n=5) [22,45,47,48,54], (3) multiple component plus an individual and group face-to-face intervention (n=1) [49], (4) mobile applications and an individual face-to-face intervention (n=2) [55,56], and (5) mobile applications and an individual plus group face-to-face intervention (n=1) [46].

In asthma studies, 2 blended self-management intervention combinations were discussed: (1) mobile application and individual face-to-face intervention (n=3) [23,58,60] and (2) internet-assisted intervention and the group face-to-face intervention (n=4) [25,59,61,62]. Detailed information on the interventions in the COPD and asthma studies is shown in Table 3.

**Table 3:** Description of the blended self-management interventions in chronic obstructive pulmonary disease and asthma studies.

COPD^a^ and asthma study			eHealth					Face-to-face		
			Category (details)		Functionality		Category (details)			Functionality
**COPD (included in the meta-analysis)**										
	Bentley et al [45]	IA^b^ (telehealth-supported service)		Guide, remind, and record		Individual (home visits)			Training	
	Chau et al [48]	IA (peripheral devices+mobile phone)		Guide, record, remind, and display		Individual (home visits)			Education and consultation	
	Garcia et al [22]	IA (web-based call centre)		Guide, remind, and record		Individual (SC^c^ and home visits)			Assessment, education, and consultation	
	Jehn et al [50]	MC^d^ (peripheral devices+mobile)		Display, record, and remind		Individual (outpatient visits)			Training, and monitoring	
	Koff et al [24]	MC (peripheral devices+web platform+phone call)		Record, display, instruct, guide, remind, and communication		Individual (home visits)			Education, consultation; training, and assessment	
	Nguyen et al [51]	MC (web modules+PDA^e^)		Guide, remind, record, and communication		Individual (home and PC^f^ visits)			Education, training, and assessment	
	Stamenova et al [53]	MC (peripheral devices+web platform+phone call)		Display, record, remind, guide, and communication		Individual (SC visits)			Assessment and consultation	
	Wang et al [54]	IA (web platform)		Guide, record, instruct, and communication		Individual (SC visit)			Monitoring	
	Wang et al [55]	MA^g^ (web-based app)		Guide and communication		Individual (SC visits)			Education	
	Wei et al [56]	MA (phone call)		Guide, remind, record, and communication		Individual (PC visits)			Education, training, and assessment	
	Xin et al [57]	MC (phone call+web platform)		Guide, record, instruct, and communication		Individual (SC visits)			Education and training	
**COPD (not included in the meta-analysis)**										
	Cameron et al [46]	MA (phone call)		Guide and communication		Individual+group (exercise guidance)			Education and consultation	
	Casas et al [47]	IA (web-based app)		Display and record		Individual (SC and home visits)			Assessment, education, and consultation	
	Haesum et al [49]	MC (peripheral devices+web platform)		Guide, record, remind, and communication		Individual+group visits			Training and monitoring	
	Sorknaes et al [52]	MC (peripheral devices+web platform)		Guide, instruct, and communication		Individual (PC visits)			Consultation	
**Asthma** **(included in the meta-analysis)**										
	Cao et al [58]	MA (Wechat app)		Guide, remind, and communication		Individual (SC visit)			Education	
	Ostojic et al [60]	MA (SMS)		Guide, display, record, and communication		Individual (PC visits)			Education	
	Türk et al [61]	IA (web platform)		Instruct, record, and communication		Group (unclear)			Education and training	
	van der Meer et al [25]	IA (web platform)		Guide, remind, record, and communication		Group (unclear)			Assessment and education	
	van Gaalen et al [62]	IA (web platform)		Guide, remind, and communication		Group (unclear)			Education and consultation	
**Asthma (not included in the meta-analysis)**										
	Barbanel et al [23]	MA (phone call)		Guide, remind, and record		Individual (unclear)			Education	
	Kohler et al [59]	IA (web platform)		Guide, record, and communication		Group (unclear)			Education and training	

^a^COPD: chronic obstructive pulmonary disease.

^b^IA: internet-assisted.

^c^SC: secondary care.

^d^MC: multiple component.

^e^PDA: personal digital assistant.

^f^PC: primary care.

^g^MA: mobile application.

#### BCTs of the Blended Self-Management Intervention

In COPD studies, the number of BCTs used in the interventions ranged from 3 to 10, with a mean of 6.42 (SD 1.99). *General information*, *Provide feedback on performance*, *Prompt self-monitoring/tracking,* and *Problem-solving/barrier* were included in 15 studies [22,24,45-57]. *Action planning* [22,46,47,51-54,56,57] and *Motivational approach* [22,24,46,47,50-52,54,55] were included in 9 studies, respectively. *Prompt review of behavioural goals* were included in 7 studies [22,46,47,49,51,53,54]. *Goal setting* was used in 6 studies [22,46,47,51,53,54]. *Social support* was reported in 4 studies [22,47,51,55], and *Emotional control training* was used in 2 studies [46,51].

In asthma studies, the number of BCTs ranged from 4 to 10, with a mean of 6.29 (SD 2.63). *General information*, *Prompt self-monitoring/ tracking*, and *Problem-solving/barrier* were used in all 7 studies [23,25,58-62]. *Provide feedback on performance* was used in 6 studies [25,58-62]. *Action planning* and *Motivational approach* were used in 4 studies [23,25,61,62]. *Goal setting* and *Prompt review of behavioural goals* were used in 3 studies [25,61,62], *Social support* was used in 2 studies [61,62], and *Emotional control training* was used in 1 study [61] (Multimedia Appendix 3 [22-25,45-62])

### Effects of the Interventions

#### Systematic Review

In COPD studies, the following 3 health-related effectiveness outcomes were reported: mortality [45,47,52], exacerbation frequency [50,57], and BMI [22]. Regarding outcome mortality, none of the 3 studies reported any effect [45,47,52]. Regarding outcome exacerbation frequency, both studies [50,57] found that the blended self-management intervention reduced the exacerbation frequency (RR=0.38; 95% CI 0.26-0.56). A study on BMI reported that blended self-management intervention had a significant effect on BMI (*d*=0.81; 95% CI 0.25-1.34) [22]. Moreover, 11 different process outcomes were studied: number of visits (including home visits, PC visits, and SC visits; n=3) [47,48,50], satisfaction with the intervention (n=3) [22,24,48], medication adherence (n=3) [22,56,57], costs (n=2) [24,45], smoking (n=2) [22,46], self-management ability (n=2) [51,55], physical activity (n=2) [22,51], nutrition (n=1) [46], alcohol (n=1) [46], psychosocial management (n=1) [46], and symptom management (n=1) [46]. Of the 3 studies, 2 showed a moderate effect (*d*=0.73; 95% CI 0.50-0.96) [56,57], whereas the other study reported a mixed effect on medication adherence [22]. Regarding the outcome self-management ability, 1 reported a large effect (*d*=1.15; 95% CI 0.66-1.62) [55], and the other study showed no effect [51]. No effect was found on the other process outcome indicators. In asthma studies, 4 health-related effectiveness outcomes were reported: admission rate [60], BMI [61], exercise capacity [61], and exacerbation frequency [25]. No effect was found on the admission rate and exacerbation frequency. A large effect was found in BMI (*d*=1.42; 95% CI 0.28-2.42) and exercise capacity (*d*=1.50; 95% CI 0.35-2.50). Three process outcomes were reported: asthma knowledge (n=2) [25,59], visits (n=2) [25,60], and adherence (therapy and medication adherence; n=2) [25,60]. No effect was found on any of the process outcome indicators.

#### Meta-analysis

**Figure 2:**
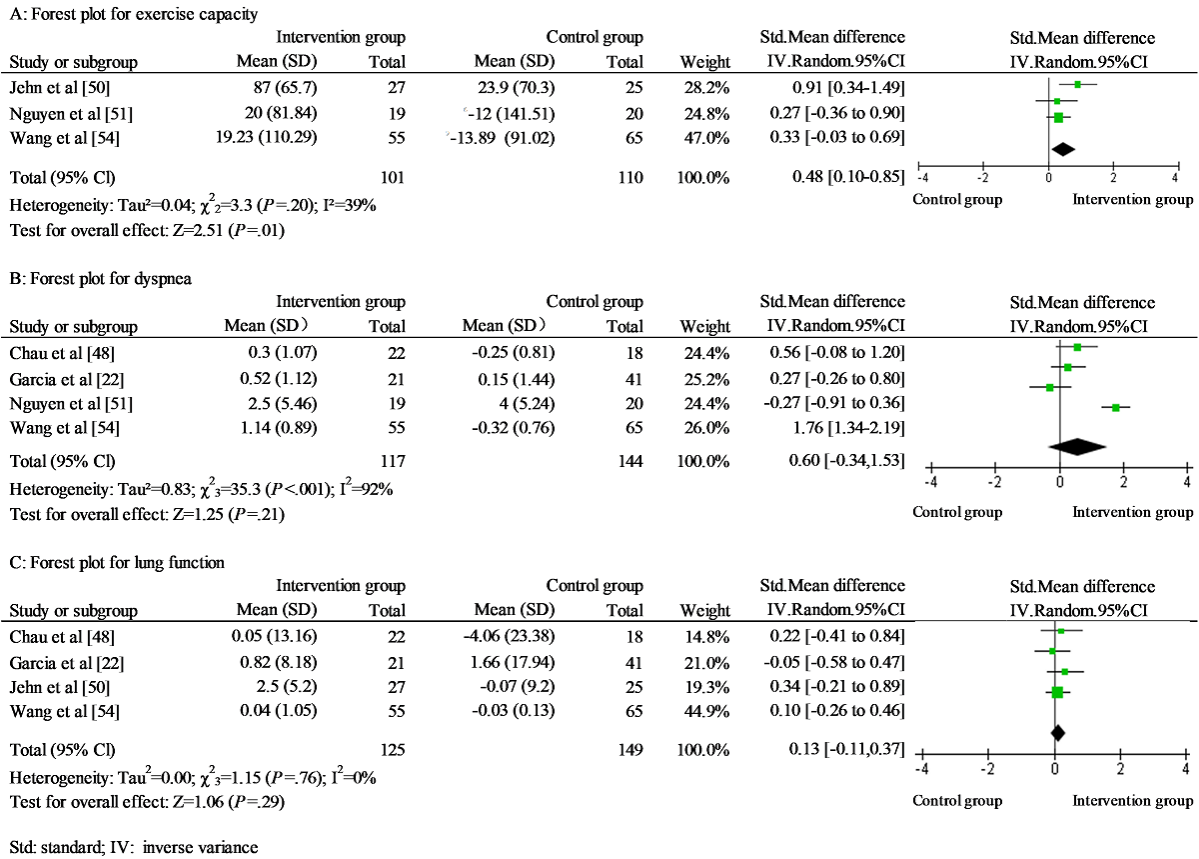
Forest plots for (A) exercise capacity, (B) dyspnea, and (C) lung function in chronic obstructive pulmonary disease studies.

**Figure 3:**
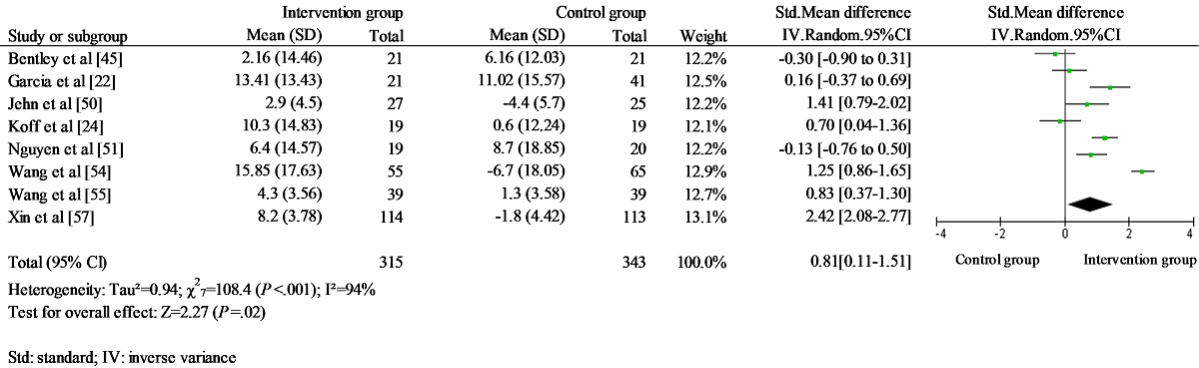
Forest plot for quality of life in chronic obstructive pulmonary disease studies.

**Figure 4:**
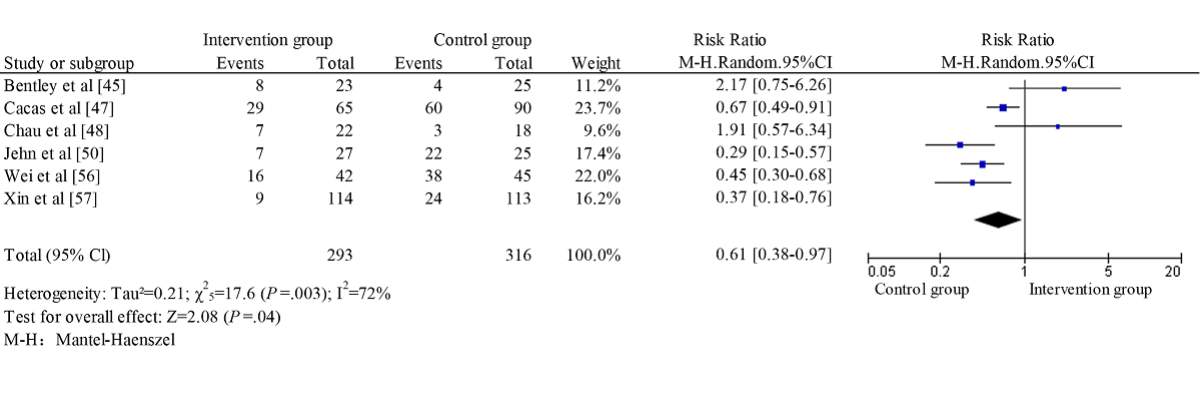
Forest plot for admission rate in chronic obstructive pulmonary disease studies.

A total of 11 studies focusing on patients with COPD were included in the meta-analysis [22,24,45,48,50,51,53-57]. The following health-related effectiveness outcomes were included: exercise capacity, dyspnea, lung function, QoL, and admission rate. Three studies reported walking distance as an indicator of exercise capacity [50,51,54]. Blended self-management intervention showed a small effect on the walking distance without significant heterogeneity (SMD*=*0.48; 95% CI 0.10-0.85, *χ*^2^_2=_3.3; *P*=.20; *I*^2^=39%; Figure 2). No study was identified as an outlier. Dyspnea was reported in 4 studies [22,48,51,54]. It was measured using the dyspnea subscale of the CRQ [48,51], Medical Research Council [22], and the Modified Medical Research Council [54]. Lung function was measured with FEV1% [48,50,54] and FEV1/FVC (%) [22] in 4 studies. No significant difference was found in dyspnea and lung function between the IG and CG (Figure 2). No study was identified as an outlier. QoL was reported in 8 studies with SGRQ [22,24,45,54], CAT [50,55,57], and CRQ [51]. A large effect was found on QoL, with substantial heterogeneity (SMD*=*0.81; 95% CI 0.11-1.51; *χ*^2^_7_=108.4; *P*<.001; *I*^2^=94% Figure 3). The standardized residual identified 1 study as an outlier [22]. Removal of this study resulted in an increased effect size without decreasing heterogeneity (SMD*=*0.90; 95% CI 0.15-1.65; *χ*^2^_6_=94.1; *P*<.001; *I*^2^=94%). Furthermore, blended self-management intervention reduced admission rate with a substantial heterogeneity (RR=0.61; 95% CI 0.38-0.97; *χ*^2^_5_=17.6; *P*=.003; *I*^2^=72%; Figure 4). No outliers were identified.

**Figure 5:**
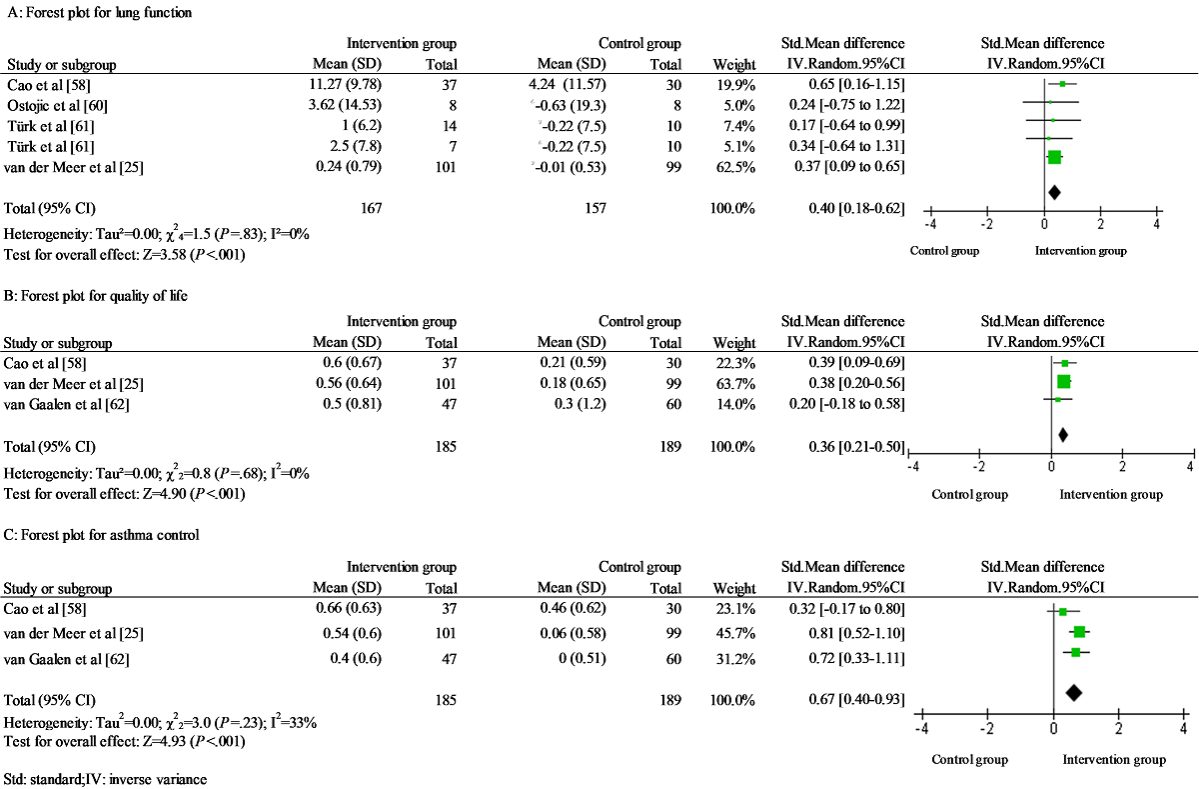
Forest plots for (A) lung function, (B) quality of life, and (C) asthma control in asthma studies.

A total of 5 asthma studies were pooled in the meta-analysis [25,58,60-62]. In addition, 3 health-related effectiveness outcomes were included: lung function, QoL, and asthma control. Lung function was reported as FEV1 (%) [58,61] and FEV_1_ [25]. Blended self-management intervention showed a small effect on the lung function without significant heterogeneity (SMD*=*0.40; 95% CI 0.18-0.62; *χ*^2^_4_=1.5; *P*=.83; *I*^2^=0%). No study was identified as an outlier. Three studies reported QoL using an asthma QoL questionnaire [25,58,62]. There was a small effect size of the blended self-management intervention on QoL without significant heterogeneity (SMD*=*0.36; 95% CI 0.21-0.50; *χ*^2^_2_=0.8; *P*=.68; *I*^2^=0%). No study was identified as an outlier. Furthermore, 3 studies reported asthma control using an asthma control questionnaire [25,58,62]. A moderate effect was found in the blended intervention self-management group without significant heterogeneity (SMD=0.67; 95% CI 0.40-0.93; *χ*^2^_2_=3.0; *P*=.23; *I*^2^=33%; Figure 5). No study was identified as an outlier.

## Discussion

### Principal Findings

This systematic review and meta-analysis assessed the effectiveness of blended self-management interventions on health-related effectiveness and process outcome indicators in patients with COPD or asthma. Of the 22 studies that were included in the systematic review, 15 were about COPD and 7 were about asthma.

Studies focusing on COPD patients included 3 different health-related effectiveness outcome indicators, and mixed effects were observed. No effect was observed on mortality. A positive effect was observed for exacerbation frequency and BMI. In total, 11 different process outcome indicators were studied (eg, medication adherence and self-management ability). Of the 3 studies, 2 reported a moderate effect on adherence. A positive effect was found in 1 of the 2 studies on self-management ability. No effects were found on the other process outcomes. Eleven COPD studies were included in the meta-analysis. Blended self-management interventions did not have a significant effect on dyspnea or lung function. Still, they did result in a small improvement in exercise capacity and a moderate improvement in QoL and decreased the admission rate. Overall, the majority of studies had some concerns about the ROB assessment.

The asthma studies included 4 health-related effectiveness outcomes. Large effects were observed in BMI and exercise capacity. There was no effect on the admission rate and exacerbation frequency. Three process outcomes were studied (ie, visits, intervention and medication adherence, and asthma knowledge). No effect was found on any of the process outcomes. Five asthma studies were included in the meta-analysis. Blended self-management intervention showed a small effect on lung function and QoL, and a moderate effect was found on asthma control. Half of the studies reported some concerns, whereas others showed a high ROB assessment.

The meta-analysis suggested that blended self-management interventions can effectively improve the exercise capacity of patients with COPD. This result was in line with another systematic review that examined the effect of COPD disease management programs, including eHealth and face-to-face components [64]. However, this finding was not consistent with a systematic review of the effect of telehealth in patients with COPD [65]. This may be because the blended programs, contrary to the telehealth programs, were likely to promote exercise capacity using various BCTs, including providing information and instruction on the behavior, self-monitoring, and providing feedback on performance by eHealth and face-to-face intervention [64]. This meta-analysis also showed that blended self-management interventions had a positive effect on QoL, which was in line with the findings of a meta-analysis that investigated the effect of COPD self-management interventions, including various self-management programs [66]. Blended self-management intervention significantly decreased admission rates. This finding was consistent with a previous meta-analysis [67], in which the effect of integrated care from health care providers with or without eHealth was identified. This might be because patients increased their self-management ability and acted on exacerbations more promptly if they received self-management intervention with multiple BCTs [68]. However, the blended self-management interventions included in this meta-analysis did not improve dyspnea and lung function, which was consistent with earlier systematic reviews that investigated the implementation of eHealth or manual therapy in patients with COPD [69,70].

Blended self-management intervention showed an inconsistent impact on process outcomes in patients with COPD. To illustrate, internet-assisted eHealth and individual face-to-face intervention showed a positive effect on self-management ability [54], whereas no effect was found in the blended intervention, including multiple eHealth components and individual face-to-face intervention [51]. The findings in this study may show that certain combinations within the blended interventions may be more effective in some outcomes; however, more large-scale studies using different combinations are needed to provide insight into this. There are several potential explanations for the lack of effects in COPD studies included in the systematic review. First, the length of the blended interventions varied among the included studies (ie, ranged from 4 to 48 weeks). The short intervention duration might have been problematic because patients with mild to very severe COPD were included in the studies. Airway obstruction is usually irreversible in those patients, and the duration of the blended interventions might have been too short to accommodate a change in health [71]. Furthermore, it appears that patients did not adhere sufficiently to blended interventions [22]. This lack of adherence might be because eHealth apps are unfamiliar to some patients [18]. We recommend that future studies educate patients on how to use eHealth because eHealth has a positive effect on improving medication adherence [72].

In asthma studies, in line with other systematic reviews focusing on integrated asthma management (ie, the cooperation of community pharmacists and general practitioners or eHealth and face-to-face intervention), the blended interventions had a positive effect on QoL and asthma control [73,74]. A previous review focusing on face-to-face interventions in patients with asthma showed that face-to-face intervention did not improve QoL and asthma control [75]. The possible reasons for this improvement could be attributed to the integrated care provided by health care providers. Health care providers can update and refer patients for education, counseling, and guidance with eHealth and face-to-face interventions [73,74]. This suggests that, compared with face-to-face interventions, blended interventions or integrated asthma management—where health care providers could refer patients for additional education, counseling, and guidance with eHealth and face-to-face intervention—are more effective. A positive effect was observed on the lung function. This finding was consistent with a meta-analysis that focused on aerobic exercise in patients with asthma [76]. This may be because adequate exercise training is beneficial to lung function. However, due to the limited number of studies included in the meta-analysis, more studies are needed to identify this effect. In this systematic review, limited studies have investigated the effects of blended interventions in patients with asthma. Therefore, the findings should be interpreted cautiously, and future studies with larger sample sizes are needed.

### Strengths and Limitations

Several strengths of this review are worth mentioning. First, a detailed description of the interventions was provided, and a wide range of outcomes was included. The detailed information might provide a helpful direction for the development of effective blended self-management interventions. Second, GRADE was used to assess the quality of evidence regarding the true effect of the blended intervention on patients with COPD and asthma. This quality of evidence assessment could provide a clear and pragmatic interpretation of the recommendations for clinicians and policy makers. Finally, we followed a strict study design and precise data analysis steps. By using a strict and precise process, we wanted to ensure the quality of the systematic review and meta-analysis.

However, several limitations also need to be addressed. First, there was a diversity in the intervention and outcome measurements, which made it difficult to compare the findings. Consequently, there may be statistical heterogeneity in the true effect size. Significant heterogeneity potentially diluted the intervention effect [77]. Second, only a small number of studies reported the same outcome measure, and studies with a small sample size were included. These studies may be underpowered to detect a true effect, and this negatively impacted the validity of these studies. Third, the quality of the evidence ranged from very low to high for all outcomes. The various quality of evidence in the outcomes may weaken the recommendation level for clinicians and researchers because the high heterogeneity among studies downgraded the quality of evidence. Fourth, we were not able to assess the risk of publication bias in the meta-analysis because few studies reported on the same outcome [40]. There may be a potential risk of publication bias. Finally, not all studies reported a follow-up. The lack of this reporting made it impossible to examine the long-term intervention effect in a comprehensive way. The results should be interpreted with caution owing to the limitations mentioned above. Larger RCTs are required to provide more insights, especially RCTs examining the effects of blended interventions in patients with asthma. Moreover, data reporting should be performed in an exact, standardized format to enable reliable extraction for future meta-analysis studies.

### Conclusions

The studies focusing on COPD found mixed effects of blended self-management interventions on health-related outcomes, with the strongest evidence found for exercise capacity, QoL, and admission rate. In asthma studies, small to moderate effects were found on asthma control, lung function, and QoL. Overall, blended self-management interventions potentially improve health-related outcomes in patients with COPD and asthma, and more studies are needed to evaluate their effectiveness.

## Appendix

Multimedia Appendix 1 Search terms.

Multimedia Appendix 2 Grading of Recommendations, Assessment, Development, and Evaluation evidence tables.

Multimedia Appendix 3 Behavior change techniques in the blended self-management interventions.

## Abbreviations

BCT: behavior change technique
CAT: chronic obstructive pulmonary disease assessment test
CG: control group
CONSORT: Consolidated Standards of Reporting Trials
COPD: chronic obstructive pulmonary disease
CRQ: chronic respiratory questionnaire
DALY: disability-adjusted life year
FEV1: forced expiratory volume in 1s
FVC: forced vital capacity
GOLD: Global Initiative for Chronic Obstructive Lung Disease
GRADE: Grading of Recommendations, Assessment, Development, and Evaluation
IG: intervention group
PC: primary care
PRISMA: Preferred Reporting Items for Systematic Reviews and Meta-Analyses
QALY: quality-adjusted life year
QoL: quality of life
RCT: randomized controlled trial
ROB: risk of bias
RR: relative ratio
SC: secondary care
SGRQ: Saint-George’s Respiratory Questionnaire
SMD: standardized mean difference
UC: usual care
