# Supplementary material for: Blended Self-Management Interventions to Reduce Disease Burden in Patients With Chronic Obstructive Pulmonary Disease and Asthma: Systematic Review and Meta-analysis
Source: J Med Internet Res. 2021 Mar 31;23(3):e24602. doi: 10.2196/24602 (PMC8047793; doi:10.2196/24602)
Supplement: Multimedia Appendix 1 [file jmir_v23i3e24602_app1.docx]

**Multimedia Appendix 1: Search terms.**

**PubMed**

(("Pulmonary Disease, Chronic Obstructive"[mesh] OR "Asthma"[mesh] OR "COPD"[tw] OR "Chronic Obstructive Lung Diseases"[tw] OR "Chronic Obstructive Lung Disease"[tw] OR "Chronic Obstructive Pulmonary Diseases"[tw] OR "Chronic Obstructive Pulmonary Disease"[tw] OR "Asthma"[mesh] OR "asthma"[tw] OR asthma*[tw] OR "Respiratory Tract Diseases"[mesh] OR lung disease*[tw] OR lung disorder*[tw] OR bronchial disease*[tw] OR bronchial disorder*[tw] OR respiratory disease*[tw] OR respiratory disorder*[tw] OR pulmonary disease*[tw] OR pulmonary disorder*[tw] OR "lung"[ti] OR "respiratory"[ti] OR "pulmonary"[ti] OR "bronchial"[ti]) AND ("Internet"[Mesh] OR "Telemedicine"[Mesh] OR web portal*[tw] OR e-consult*[tw] OR econsult*[tw] OR telemed*[tw] OR "ehealth"[tw] OR "e-health"[tw] OR "mhealth"[tw] OR "m-health"[tw] OR "mobile health"[tw] OR "telehealth"[tw] OR electronic communication*[tw] OR "remote communication"[tw] OR "remote computer"[tw] OR "remote computers"[tw] OR "remote consultation"[tw] OR "remote health care"[tw] OR "remote healthcare"[tw] OR "remote monitoring"[tw] OR "remote system"[tw] OR "remote systems"[tw] OR "remote technologies"[tw] OR "remote technology"[tw] OR "teleconsultation"[tw] OR teleconsult*[tw] OR mobile*[tw] OR "webbased"[tw] OR "web-based"[tw] OR "Cell Phones"[tw] OR "Smartphone"[tw] OR "Text Messaging"[tw] OR "Cell Phone"[tw] OR "Smartphones"[tw] OR iphon*[tw] OR "Text Messaging"[tw] OR text messag*[tw] OR "texting"[tw] OR "Electronic Mail"[mesh] OR "Electronic Mail"[tw] OR e-mail*[tw] OR email*[tw] OR "Telecommunications"[mesh:noexp] OR "app"[tw] OR "apps"[tw] OR webapp*[tw] OR "SMS"[tw] OR "mass communication"[tw] OR "blogging"[tw] OR "blog"[tw] OR "weblog"[tw] OR "social media"[tw] OR twitter*[tw] OR facebook*[tw] OR webcast*[tw] OR "Webcasts as Topic"[mesh] OR "Cell Phones"[tw] OR "Smartphone"[tw] OR "Text Messaging"[tw] OR "Cell Phone"[tw] OR "Smartphones"[tw] OR iphon*[tw] OR "Text Messaging"[tw] OR text messag*[tw] OR "texting"[tw] OR "Electronic Mail"[mesh] OR "Electronic Mail"[tw] OR e-mail*[tw] OR email*[tw] OR "app"[tw] OR "apps"[tw] OR webapp*[tw] OR "SMS"[tw] OR "mass communication"[tw] OR "blogging"[tw] OR "blog"[tw] OR "weblog"[tw] OR "social media"[tw] OR twitter*[tw] OR facebook*[tw] OR webcast*[tw] OR "Webcasts as Topic"[mesh] OR "information technology"[tw] OR "mobile"[tw] OR "cellular phone"[tw] OR "smart phone"[tw] OR "telemedicine"[tw] OR "tele-care"[tw] OR "telecare"[tw] OR "tele-monitoring"[tw] OR "telemonitoring"[tw] OR "website"[tw] OR "websites"[tw] OR "wireless"[tw] OR "personal digital assistant"[tw] OR "computer-assisted instruction"[tw] OR "social network"[tw] OR social network*[tw] OR "ipad"[tw] OR ipad*[tw] OR "telenursing"[tw] OR telenurs*[tw] OR "virtual community"[tw] OR "webpage"[tw] OR "webpages"[tw] OR "web application"[tw] OR "web applications"[tw] OR "web access"[tw] OR "teleconference"[tw] OR teleconferen*[tw] OR telecommunication*[tiab] OR "short message service"[tw] OR "Internet"[mesh] OR "internet"[tw] OR "online"[tw] OR "digital"[tw] OR digital*[tw] OR "Reminder Systems"[mesh] OR "Reminder Systems"[tw] OR "Reminder System"[tw] OR "Reminder Device"[tw] OR "Reminder Devices"[tw] OR "reminder messages"[tw] OR "reminder message"[tw] OR "web"[tw] OR "Virtual Reality"[mesh] OR "Virtual Reality"[tw] OR "Distance Counseling"[mesh] OR "Distance Counseling"[tw] OR "Distance Counselling"[tw]) AND ("Counseling"[mesh:noexp] OR "Directive Counseling"[tw] OR "Directive Counselling"[tw] OR "Counseling"[tw] OR "counselling"[tw] OR "Coaching"[tw] OR "coach"[tw] OR "coached"[tw] OR "Mentoring"[Mesh] OR "Mentoring"[tw] OR "Therapist-guided"[tw] OR "enhanced usual care"[tw] OR "nurse support"[tw] OR nurse support*[tw] OR (("Nurses"[mesh] OR "nurse"[tw] OR "nurses"[tw] OR "nursing"[tw] OR "Nursing"[mesh]) AND ("Social Support"[mesh] OR "support"[tw])) OR "guidance"[tw] OR guidanc*[tw] OR "personal coach"[tw] OR personal coach*[tw] OR "Patient Education as Topic"[Mesh:NoExp] OR "Patient Education"[tw] OR "face to face"[tw] OR "facetoface"[tw]) NOT (("Infant"[mesh] OR "Child"[mesh]) NOT "Adult"[mesh]))

**Embase**

((*"Chronic Obstructive Lung Disease"/ OR exp *"Asthma"/ OR "COPD".ti,ab OR "Chronic Obstructive Lung Diseases".ti,ab OR "Chronic Obstructive Lung Disease".ti,ab OR "Chronic Obstructive Pulmonary Diseases".ti,ab OR "Chronic Obstructive Pulmonary Disease".ti,ab OR "asthma".ti,ab OR asthma*.ti,ab OR exp *"Respiratory Tract Disease"/ OR "lung disease*".ti,ab OR "lung disorder*".ti,ab OR "bronchial disease*".ti,ab OR "bronchial disorder*".ti,ab OR "respiratory disease*".ti,ab OR "respiratory disorder*".ti,ab OR "pulmonary disease*".ti,ab OR "pulmonary disorder*".ti,ab OR "lung".ti OR "respiratory".ti OR "pulmonary".ti OR "bronchial".ti) AND (exp *"Internet"/ OR exp *"Telehealth"/ OR "web portal*".ti,ab OR "e-consult*".ti,ab OR econsult*.ti,ab OR telemed*.ti,ab OR "ehealth".ti,ab OR "e-health".ti,ab OR "mhealth".ti,ab OR "m-health".ti,ab OR "mobile health".ti,ab OR "telehealth".ti,ab OR "electronic communication*".ti,ab OR "remote communication".ti,ab OR "remote computer".ti,ab OR "remote computers".ti,ab OR "remote consultation".ti,ab OR "remote health care".ti,ab OR "remote healthcare".ti,ab OR "remote monitoring".ti,ab OR "remote system".ti,ab OR "remote systems".ti,ab OR "remote technologies".ti,ab OR "remote technology".ti,ab OR "teleconsultation".ti,ab OR teleconsult*.ti,ab OR mobile*.ti,ab OR "webbased".ti,ab OR "web-based".ti,ab OR "Cell Phones".ti,ab OR "Smartphone".ti,ab OR "Text Messaging".ti,ab OR "Cell Phone".ti,ab OR "Smartphones".ti,ab OR iphon*.ti,ab OR "Text Messaging".ti,ab OR "text messag*".ti,ab OR "texting".ti,ab OR *"e-mail"/ OR "Electronic Mail".ti,ab OR "e-mail*".ti,ab OR email*.ti,ab OR "app".ti,ab OR "apps".ti,ab OR webapp*.ti,ab OR "SMS".ti,ab OR "mass communication".ti,ab OR "blogging".ti,ab OR "blog".ti,ab OR "weblog".ti,ab OR "social media".ti,ab OR twitter*.ti,ab OR facebook*.ti,ab OR webcast*.ti,ab OR *"Webcast"/ OR "Cell Phones".ti,ab OR "Smartphone".ti,ab OR "Text Messaging".ti,ab OR "Cell Phone".ti,ab OR "Smartphones".ti,ab OR iphon*.ti,ab OR "Text Messaging".ti,ab OR "text messag*".ti,ab OR "texting".ti,ab OR "Electronic Mail".ti,ab OR e-mail*.ti,ab OR email*.ti,ab OR "app".ti,ab OR "apps".ti,ab OR webapp*.ti,ab OR "SMS".ti,ab OR "mass communication".ti,ab OR "blogging".ti,ab OR "blog".ti,ab OR "weblog".ti,ab OR "social media".ti,ab OR twitter*.ti,ab OR facebook*.ti,ab OR webcast*.ti,ab OR "information technology".ti,ab OR "mobile".ti,ab OR "cellular phone".ti,ab OR "smart phone".ti,ab OR "telemedicine".ti,ab OR "tele-care".ti,ab OR "telecare".ti,ab OR "tele-monitoring".ti,ab OR "telemonitoring".ti,ab OR "website".ti,ab OR "websites".ti,ab OR "wireless".ti,ab OR "personal digital assistant".ti,ab OR "computer-assisted instruction".ti,ab OR "social network".ti,ab OR "social network*".ti,ab OR "ipad".ti,ab OR ipad*.ti,ab OR "telenursing".ti,ab OR telenurs*.ti,ab OR "virtual community".ti,ab OR "webpage".ti,ab OR "webpages".ti,ab OR "web application".ti,ab OR "web applications".ti,ab OR "web access".ti,ab OR "teleconference".ti,ab OR teleconferen*.ti,ab OR telecommunication*.ti,ab OR "short message service".ti,ab OR "internet".ti,ab OR "online".ti,ab OR "digital".ti,ab OR digital*.ti,ab OR *"Reminder System"/ OR "Reminder Systems".ti,ab OR "Reminder System".ti,ab OR "Reminder Device".ti,ab OR "Reminder Devices".ti,ab OR "reminder messages".ti,ab OR "reminder message".ti,ab OR "web".ti,ab OR exp *"Virtual Reality"/ OR "Virtual Reality".ti,ab OR *"e-counseling"/ OR "Distance Counseling".ti,ab OR "Distance Counselling".ti,ab) AND ("Counseling"/ OR "Patient Counseling"/ OR "Directive Counseling".ti,ab OR "Directive Counselling".ti,ab OR "Counseling".ti,ab OR "counselling".ti,ab OR "Coaching".ti,ab OR "coach".ti,ab OR "coached".ti,ab OR "Mentoring"/ OR "Mentoring".ti,ab OR "Therapist-guided".ti,ab OR "enhanced usual care".ti,ab OR "nurse support".ti,ab OR "nurse support*".ti,ab OR ((exp "Nurse"/ OR "nurse".ti,ab OR "nurses".ti,ab OR "nursing".ti,ab OR exp "Nursing"/) AND (exp "Social Support"/ OR "support".ti,ab)) OR "guidance".ti,ab OR guidanc*.ti,ab OR "personal coach".ti,ab OR "personal coach*".ti,ab OR "Patient Education"/ OR "Patient Education".ti,ab OR "face to face".ti,ab OR "facetoface".ti,ab) NOT ((exp "Infant"/ OR exp "Child"/) NOT exp "Adult"/)) NOT (conference review or conference abstract).pt

**Web of Science**

(((ti=("Chronic Obstructive Lung Disease" OR "Asthma" OR "COPD" OR "Chronic Obstructive Lung Diseases" OR "Chronic Obstructive Lung Disease" OR "Chronic Obstructive Pulmonary Diseases" OR "Chronic Obstructive Pulmonary Disease" OR "asthma" OR asthma* OR "Respiratory Tract Disease" OR "lung disease" OR "lung disorder" OR "bronchial disease" OR "bronchial disorder" OR "respiratory disease" OR "respiratory disorder" OR "pulmonary disease" OR "pulmonary disorder") OR ti=("lung" OR "respiratory" OR "pulmonary" OR "bronchial")) AND ts=("Internet" OR "Telehealth" OR "web portal" OR "e-consult" OR econsult* OR telemed* OR "ehealth" OR "e-health" OR "mhealth" OR "m-health" OR "mobile health" OR "telehealth" OR "electronic communication" OR "remote communication" OR "remote computer" OR "remote computers" OR "remote consultation" OR "remote health care" OR "remote healthcare" OR "remote monitoring" OR "remote system" OR "remote systems" OR "remote technologies" OR "remote technology" OR "teleconsultation" OR teleconsult* OR mobile* OR "webbased" OR "web-based" OR "Cell Phones" OR "Smartphone" OR "Text Messaging" OR "Cell Phone" OR "Smartphones" OR iphon* OR "Text Messaging" OR "text messag" OR "texting" OR "e-mail" OR "Electronic Mail" OR "e-mail" OR email* OR "app" OR "apps" OR webapp* OR "SMS" OR "mass communication" OR "blogging" OR "blog" OR "weblog" OR "social media" OR twitter* OR facebook* OR webcast* OR "Webcast" OR "Cell Phones" OR "Smartphone" OR "Text Messaging" OR "Cell Phone" OR "Smartphones" OR iphon* OR "Text Messaging" OR "text messag" OR "texting" OR "Electronic Mail" OR e-mail* OR email* OR "app" OR "apps" OR webapp* OR "SMS" OR "mass communication" OR "blogging" OR "blog" OR "weblog" OR "social media" OR twitter* OR facebook* OR webcast* OR "information technology" OR "mobile" OR "cellular phone" OR "smart phone" OR "telemedicine" OR "tele-care" OR "telecare" OR "tele-monitoring" OR "telemonitoring" OR "website" OR "websites" OR "wireless" OR "personal digital assistant" OR "computer-assisted instruction" OR "social network" OR "social network" OR "ipad" OR ipad* OR "telenursing" OR telenurs* OR "virtual community" OR "webpage" OR "webpages" OR "web application" OR "web applications" OR "web access" OR "teleconference" OR teleconferen* OR telecommunication* OR "short message service" OR "internet" OR "online" OR "digital" OR digital* OR "Reminder System" OR "Reminder Systems" OR "Reminder System" OR "Reminder Device" OR "Reminder Devices" OR "reminder messages" OR "reminder message" OR "web" OR "Virtual Reality" OR "Virtual Reality" OR "e-counseling" OR "Distance Counseling" OR "Distance Counselling") AND ts=("Counseling" OR "Patient Counseling" OR "Directive Counseling" OR "Directive Counselling" OR "Counseling" OR "counselling" OR "Coaching" OR "coach" OR "coached" OR "Mentoring" OR "Mentoring" OR "Therapist-guided" OR "enhanced usual care" OR "nurse support" OR "nurse support" OR (("Nurse" OR "nurse" OR "nurses" OR "nursing" OR "Nursing") AND ("Social Support" OR "support")) OR "guidance" OR guidanc* OR "personal coach" OR "personal coach" OR "Patient Education" OR "Patient Education" OR "face to face" OR "facetoface") NOT ti=((Infan* OR Child* OR pediatr* OR paediatr*) NOT (Adult* OR elderl*)))) **OR** (((ts=("Chronic Obstructive Lung Disease" OR "Asthma" OR "COPD" OR "Chronic Obstructive Lung Diseases" OR "Chronic Obstructive Lung Disease" OR "Chronic Obstructive Pulmonary Diseases" OR "Chronic Obstructive Pulmonary Disease" OR "asthma" OR asthma* OR "Respiratory Tract Disease" OR "lung disease" OR "lung disorder" OR "bronchial disease" OR "bronchial disorder" OR "respiratory disease" OR "respiratory disorder" OR "pulmonary disease" OR "pulmonary disorder") OR ti=("lung" OR "respiratory" OR "pulmonary" OR "bronchial")) AND ti=("Internet" OR "Telehealth" OR "web portal" OR "e-consult" OR econsult* OR telemed* OR "ehealth" OR "e-health" OR "mhealth" OR "m-health" OR "mobile health" OR "telehealth" OR "electronic communication" OR "remote communication" OR "remote computer" OR "remote computers" OR "remote consultation" OR "remote health care" OR "remote healthcare" OR "remote monitoring" OR "remote system" OR "remote systems" OR "remote technologies" OR "remote technology" OR "teleconsultation" OR teleconsult* OR mobile* OR "webbased" OR "web-based" OR "Cell Phones" OR "Smartphone" OR "Text Messaging" OR "Cell Phone" OR "Smartphones" OR iphon* OR "Text Messaging" OR "text messag" OR "texting" OR "e-mail" OR "Electronic Mail" OR "e-mail" OR email* OR "app" OR "apps" OR webapp* OR "SMS" OR "mass communication" OR "blogging" OR "blog" OR "weblog" OR "social media" OR twitter* OR facebook* OR webcast* OR "Webcast" OR "Cell Phones" OR "Smartphone" OR "Text Messaging" OR "Cell Phone" OR "Smartphones" OR iphon* OR "Text Messaging" OR "text messag" OR "texting" OR "Electronic Mail" OR e-mail* OR email* OR "app" OR "apps" OR webapp* OR "SMS" OR "mass communication" OR "blogging" OR "blog" OR "weblog" OR "social media" OR twitter* OR facebook* OR webcast* OR "information technology" OR "mobile" OR "cellular phone" OR "smart phone" OR "telemedicine" OR "tele-care" OR "telecare" OR "tele-monitoring" OR "telemonitoring" OR "website" OR "websites" OR "wireless" OR "personal digital assistant" OR "computer-assisted instruction" OR "social network" OR "social network" OR "ipad" OR ipad* OR "telenursing" OR telenurs* OR "virtual community" OR "webpage" OR "webpages" OR "web application" OR "web applications" OR "web access" OR "teleconference" OR teleconferen* OR telecommunication* OR "short message service" OR "internet" OR "online" OR "digital" OR digital* OR "Reminder System" OR "Reminder Systems" OR "Reminder System" OR "Reminder Device" OR "Reminder Devices" OR "reminder messages" OR "reminder message" OR "web" OR "Virtual Reality" OR "Virtual Reality" OR "e-counseling" OR "Distance Counseling" OR "Distance Counselling") AND ts=("Counseling" OR "Patient Counseling" OR "Directive Counseling" OR "Directive Counselling" OR "Counseling" OR "counselling" OR "Coaching" OR "coach" OR "coached" OR "Mentoring" OR "Mentoring" OR "Therapist-guided" OR "enhanced usual care" OR "nurse support" OR "nurse support" OR (("Nurse" OR "nurse" OR "nurses" OR "nursing" OR "Nursing") AND ("Social Support" OR "support")) OR "guidance" OR guidanc* OR "personal coach" OR "personal coach" OR "Patient Education" OR "Patient Education" OR "face to face" OR "facetoface") NOT ti=((Infan* OR Child* OR pediatr* OR paediatr*) NOT (Adult* OR elderl*)))) **OR** (((ts=("Chronic Obstructive Lung Disease" OR "Asthma" OR "COPD" OR "Chronic Obstructive Lung Diseases" OR "Chronic Obstructive Lung Disease" OR "Chronic Obstructive Pulmonary Diseases" OR "Chronic Obstructive Pulmonary Disease" OR "asthma" OR asthma* OR "Respiratory Tract Disease" OR "lung disease" OR "lung disorder" OR "bronchial disease" OR "bronchial disorder" OR "respiratory disease" OR "respiratory disorder" OR "pulmonary disease" OR "pulmonary disorder") OR ti=("lung" OR "respiratory" OR "pulmonary" OR "bronchial")) AND ts=("Internet" OR "Telehealth" OR "web portal" OR "e-consult" OR econsult* OR telemed* OR "ehealth" OR "e-health" OR "mhealth" OR "m-health" OR "mobile health" OR "telehealth" OR "electronic communication" OR "remote communication" OR "remote computer" OR "remote computers" OR "remote consultation" OR "remote health care" OR "remote healthcare" OR "remote monitoring" OR "remote system" OR "remote systems" OR "remote technologies" OR "remote technology" OR "teleconsultation" OR teleconsult* OR mobile* OR "webbased" OR "web-based" OR "Cell Phones" OR "Smartphone" OR "Text Messaging" OR "Cell Phone" OR "Smartphones" OR iphon* OR "Text Messaging" OR "text messag" OR "texting" OR "e-mail" OR "Electronic Mail" OR "e-mail" OR email* OR "app" OR "apps" OR webapp* OR "SMS" OR "mass communication" OR "blogging" OR "blog" OR "weblog" OR "social media" OR twitter* OR facebook* OR webcast* OR "Webcast" OR "Cell Phones" OR "Smartphone" OR "Text Messaging" OR "Cell Phone" OR "Smartphones" OR iphon* OR "Text Messaging" OR "text messag" OR "texting" OR "Electronic Mail" OR e-mail* OR email* OR "app" OR "apps" OR webapp* OR "SMS" OR "mass communication" OR "blogging" OR "blog" OR "weblog" OR "social media" OR twitter* OR facebook* OR webcast* OR "information technology" OR "mobile" OR "cellular phone" OR "smart phone" OR "telemedicine" OR "tele-care" OR "telecare" OR "tele-monitoring" OR "telemonitoring" OR "website" OR "websites" OR "wireless" OR "personal digital assistant" OR "computer-assisted instruction" OR "social network" OR "social network" OR "ipad" OR ipad* OR "telenursing" OR telenurs* OR "virtual community" OR "webpage" OR "webpages" OR "web application" OR "web applications" OR "web access" OR "teleconference" OR teleconferen* OR telecommunication* OR "short message service" OR "internet" OR "online" OR "digital" OR digital* OR "Reminder System" OR "Reminder Systems" OR "Reminder System" OR "Reminder Device" OR "Reminder Devices" OR "reminder messages" OR "reminder message" OR "web" OR "Virtual Reality" OR "Virtual Reality" OR "e-counseling" OR "Distance Counseling" OR "Distance Counselling") AND ti=("Counseling" OR "Patient Counseling" OR "Directive Counseling" OR "Directive Counselling" OR "Counseling" OR "counselling" OR "Coaching" OR "coach" OR "coached" OR "Mentoring" OR "Mentoring" OR "Therapist-guided" OR "enhanced usual care" OR "nurse support" OR "nurse support" OR (("Nurse" OR "nurse" OR "nurses" OR "nursing" OR "Nursing") AND ("Social Support" OR "support")) OR "guidance" OR guidanc* OR "personal coach" OR "personal coach" OR "Patient Education" OR "Patient Education" OR "face to face" OR "facetoface") NOT ti=((Infan* OR Child* OR pediatr* OR paediatr*) NOT (Adult* OR elderl*))))

**Cochrane**

(("Chronic Obstructive Lung Disease" OR "Asthma" OR "COPD" OR "Chronic Obstructive Lung Diseases" OR "Chronic Obstructive Lung Disease" OR "Chronic Obstructive Pulmonary Diseases" OR "Chronic Obstructive Pulmonary Disease" OR "asthma" OR asthma* OR "Respiratory Tract Disease" OR "lung disease" OR "lung disorder" OR "bronchial disease" OR "bronchial disorder" OR "respiratory disease" OR "respiratory disorder" OR "pulmonary disease" OR "pulmonary disorder"):ti,ab,kw OR ("lung" OR "respiratory" OR "pulmonary" OR "bronchial"):ti) AND ("Internet" OR "Telehealth" OR "web portal" OR "e-consult" OR econsult* OR telemed* OR "ehealth" OR "e-health" OR "mhealth" OR "m-health" OR "mobile health" OR "telehealth" OR "electronic communication" OR "remote communication" OR "remote computer" OR "remote computers" OR "remote consultation" OR "remote health care" OR "remote healthcare" OR "remote monitoring" OR "remote system" OR "remote systems" OR "remote technologies" OR "remote technology" OR "teleconsultation" OR teleconsult* OR mobile* OR "webbased" OR "web-based" OR "Cell Phones" OR "Smartphone" OR "Text Messaging" OR "Cell Phone" OR "Smartphones" OR iphon* OR "Text Messaging" OR "text messag" OR "texting" OR "e-mail" OR "Electronic Mail" OR "e-mail" OR email* OR "app" OR "apps" OR webapp* OR "SMS" OR "mass communication" OR "blogging" OR "blog" OR "weblog" OR "social media" OR twitter* OR facebook* OR webcast* OR "Webcast" OR "Cell Phones" OR "Smartphone" OR "Text Messaging" OR "Cell Phone" OR "Smartphones" OR iphon* OR "Text Messaging" OR "text messag" OR "texting" OR "Electronic Mail" OR e-mail* OR email* OR "app" OR "apps" OR webapp* OR "SMS" OR "mass communication" OR "blogging" OR "blog" OR "weblog" OR "social media" OR twitter* OR facebook* OR webcast* OR "information technology" OR "mobile" OR "cellular phone" OR "smart phone" OR "telemedicine" OR "tele-care" OR "telecare" OR "tele-monitoring" OR "telemonitoring" OR "website" OR "websites" OR "wireless" OR "personal digital assistant" OR "computer-assisted instruction" OR "social network" OR "social network" OR "ipad" OR ipad* OR "telenursing" OR telenurs* OR "virtual community" OR "webpage" OR "webpages" OR "web application" OR "web applications" OR "web access" OR "teleconference" OR teleconferen* OR telecommunication* OR "short message service" OR "internet" OR "online" OR "digital" OR digital* OR "Reminder System" OR "Reminder Systems" OR "Reminder System" OR "Reminder Device" OR "Reminder Devices" OR "reminder messages" OR "reminder message" OR "web" OR "Virtual Reality" OR "Virtual Reality" OR "e-counseling" OR "Distance Counseling" OR "Distance Counselling"):ti,ab,kw AND ("Counseling" OR "Patient Counseling" OR "Directive Counseling" OR "Directive Counselling" OR "Counseling" OR "counselling" OR "Coaching" OR "coach" OR "coached" OR "Mentoring" OR "Mentoring" OR "Therapist-guided" OR "enhanced usual care" OR "nurse support" OR "nurse support" OR (("Nurse" OR "nurse" OR "nurses" OR "nursing" OR "Nursing") AND ("Social Support" OR "support")) OR "guidance" OR guidanc* OR "personal coach" OR "personal coach" OR "Patient Education" OR "Patient Education" OR "face to face" OR "facetoface"):ti,ab,kw NOT ((Infan* OR Child* OR pediatr* OR paediatr*) NOT (Adult* OR elderl*)):ti

**Emcare**

((*"Chronic Obstructive Lung Disease"/ OR exp *"Asthma"/ OR "COPD".ti,ab OR "Chronic Obstructive Lung Diseases".ti,ab OR "Chronic Obstructive Lung Disease".ti,ab OR "Chronic Obstructive Pulmonary Diseases".ti,ab OR "Chronic Obstructive Pulmonary Disease".ti,ab OR "asthma".ti,ab OR asthma*.ti,ab OR exp *"Respiratory Tract Disease"/ OR "lung disease*".ti,ab OR "lung disorder*".ti,ab OR "bronchial disease*".ti,ab OR "bronchial disorder*".ti,ab OR "respiratory disease*".ti,ab OR "respiratory disorder*".ti,ab OR "pulmonary disease*".ti,ab OR "pulmonary disorder*".ti,ab OR "lung".ti OR "respiratory".ti OR "pulmonary".ti OR "bronchial".ti) AND (exp *"Internet"/ OR exp *"Telehealth"/ OR "web portal*".ti,ab OR "e-consult*".ti,ab OR econsult*.ti,ab OR telemed*.ti,ab OR "ehealth".ti,ab OR "e-health".ti,ab OR "mhealth".ti,ab OR "m-health".ti,ab OR "mobile health".ti,ab OR "telehealth".ti,ab OR "electronic communication*".ti,ab OR "remote communication".ti,ab OR "remote computer".ti,ab OR "remote computers".ti,ab OR "remote consultation".ti,ab OR "remote health care".ti,ab OR "remote healthcare".ti,ab OR "remote monitoring".ti,ab OR "remote system".ti,ab OR "remote systems".ti,ab OR "remote technologies".ti,ab OR "remote technology".ti,ab OR "teleconsultation".ti,ab OR teleconsult*.ti,ab OR mobile*.ti,ab OR "webbased".ti,ab OR "web-based".ti,ab OR "Cell Phones".ti,ab OR "Smartphone".ti,ab OR "Text Messaging".ti,ab OR "Cell Phone".ti,ab OR "Smartphones".ti,ab OR iphon*.ti,ab OR "Text Messaging".ti,ab OR "text messag*".ti,ab OR "texting".ti,ab OR *"e-mail"/ OR "Electronic Mail".ti,ab OR "e-mail*".ti,ab OR email*.ti,ab OR "app".ti,ab OR "apps".ti,ab OR webapp*.ti,ab OR "SMS".ti,ab OR "mass communication".ti,ab OR "blogging".ti,ab OR "blog".ti,ab OR "weblog".ti,ab OR "social media".ti,ab OR twitter*.ti,ab OR facebook*.ti,ab OR webcast*.ti,ab OR *"Webcast"/ OR "Cell Phones".ti,ab OR "Smartphone".ti,ab OR "Text Messaging".ti,ab OR "Cell Phone".ti,ab OR "Smartphones".ti,ab OR iphon*.ti,ab OR "Text Messaging".ti,ab OR "text messag*".ti,ab OR "texting".ti,ab OR "Electronic Mail".ti,ab OR e-mail*.ti,ab OR email*.ti,ab OR "app".ti,ab OR "apps".ti,ab OR webapp*.ti,ab OR "SMS".ti,ab OR "mass communication".ti,ab OR "blogging".ti,ab OR "blog".ti,ab OR "weblog".ti,ab OR "social media".ti,ab OR twitter*.ti,ab OR facebook*.ti,ab OR webcast*.ti,ab OR "information technology".ti,ab OR "mobile".ti,ab OR "cellular phone".ti,ab OR "smart phone".ti,ab OR "telemedicine".ti,ab OR "tele-care".ti,ab OR "telecare".ti,ab OR "tele-monitoring".ti,ab OR "telemonitoring".ti,ab OR "website".ti,ab OR "websites".ti,ab OR "wireless".ti,ab OR "personal digital assistant".ti,ab OR "computer-assisted instruction".ti,ab OR "social network".ti,ab OR "social network*".ti,ab OR "ipad".ti,ab OR ipad*.ti,ab OR "telenursing".ti,ab OR telenurs*.ti,ab OR "virtual community".ti,ab OR "webpage".ti,ab OR "webpages".ti,ab OR "web application".ti,ab OR "web applications".ti,ab OR "web access".ti,ab OR "teleconference".ti,ab OR teleconferen*.ti,ab OR telecommunication*.ti,ab OR "short message service".ti,ab OR "internet".ti,ab OR "online".ti,ab OR "digital".ti,ab OR digital*.ti,ab OR *"Reminder System"/ OR "Reminder Systems".ti,ab OR "Reminder System".ti,ab OR "Reminder Device".ti,ab OR "Reminder Devices".ti,ab OR "reminder messages".ti,ab OR "reminder message".ti,ab OR "web".ti,ab OR exp *"Virtual Reality"/ OR "Virtual Reality".ti,ab OR *"e-counseling"/ OR "Distance Counseling".ti,ab OR "Distance Counselling".ti,ab) AND ("Counseling"/ OR "Patient Counseling"/ OR "Directive Counseling".ti,ab OR "Directive Counselling".ti,ab OR "Counseling".ti,ab OR "counselling".ti,ab OR "Coaching".ti,ab OR "coach".ti,ab OR "coached".ti,ab OR "Mentoring"/ OR "Mentoring".ti,ab OR "Therapist-guided".ti,ab OR "enhanced usual care".ti,ab OR "nurse support".ti,ab OR "nurse support*".ti,ab OR ((exp "Nurse"/ OR "nurse".ti,ab OR "nurses".ti,ab OR "nursing".ti,ab OR exp "Nursing"/) AND (exp "Social Support"/ OR "support".ti,ab)) OR "guidance".ti,ab OR guidanc*.ti,ab OR "personal coach".ti,ab OR "personal coach*".ti,ab OR "Patient Education"/ OR "Patient Education".ti,ab OR "face to face".ti,ab OR "facetoface".ti,ab) NOT ((exp "Infant"/ OR exp "Child"/) NOT exp "Adult"/)) NOT (conference review or conference abstract).pt

Search terms related to COPD or asthma, and blended intervention

**PubMed**

(("Pulmonary Disease, Chronic Obstructive"[mesh] OR "Asthma"[mesh] OR "COPD"[tw] OR "Chronic Obstructive Lung Diseases"[tw] OR "Chronic Obstructive Lung Disease"[tw] OR "Chronic Obstructive Pulmonary Diseases"[tw] OR "Chronic Obstructive Pulmonary Disease"[tw] OR "Asthma"[mesh] OR "asthma"[tw] OR asthma*[tw] OR "Respiratory Tract Diseases"[mesh] OR lung disease*[tw] OR lung disorder*[tw] OR bronchial disease*[tw] OR bronchial disorder*[tw] OR respiratory disease*[tw] OR respiratory disorder*[tw] OR pulmonary disease*[tw] OR pulmonary disorder*[tw] OR "lung"[ti] OR "respiratory"[ti] OR "pulmonary"[ti] OR "bronchial"[ti]) AND ("blended intervention"[tw] OR "blended interventions"[tw] OR "blended care"[tw] OR "blended approach"[tw] OR "blended approaches"[tw] OR "blended learning"[tw] OR (("blending"[tw] OR "blended"[tw]) AND (intervention*[tw] OR "care"[tw] OR "healthcare"[tw] OR multidisciplin*[tw] OR "learning"[tw]))) NOT (("Infant"[mesh] OR "Child"[mesh]) NOT "Adult"[mesh]))

**Embase**

(("Chronic Obstructive Lung Disease"/ OR exp "Asthma"/ OR "COPD".mp OR "Chronic Obstructive Lung Diseases".mp OR "Chronic Obstructive Lung Disease".mp OR "Chronic Obstructive Pulmonary Diseases".mp OR "Chronic Obstructive Pulmonary Disease".mp OR "asthma".mp OR asthma*.mp OR exp "Respiratory Tract Disease"/ OR "lung disease*".mp OR "lung disorder*".mp OR "bronchial disease*".mp OR "bronchial disorder*".mp OR "respiratory disease*".mp OR "respiratory disorder*".mp OR "pulmonary disease*".mp OR "pulmonary disorder*".mp OR "lung".ti OR "respiratory".ti OR "pulmonary".ti OR "bronchial".ti) AND ("blended intervention".mp OR "blended interventions".mp OR "blended care".mp OR "blended approach".mp OR "blended approaches".mp OR "blended learning".mp OR (("blending".mp OR "blended".mp) AND ("intervention".mp OR "interventions".mp OR "care".ti,ab OR "healthcare".ti,ab OR multidisciplin*.mp OR "learning".mp))) NOT ((exp "Infant"/ OR exp "Child"/) NOT exp "Adult"/)) NOT (conference review or conference abstract).pt

**Web of Science**

TS=(("Chronic Obstructive Lung Disease" OR "Asthma" OR "COPD" OR "Chronic Obstructive Lung Diseases" OR "Chronic Obstructive Lung Disease" OR "Chronic Obstructive Pulmonary Diseases" OR "Chronic Obstructive Pulmonary Disease" OR "asthma" OR asthma* OR "Respiratory Tract Disease" OR "lung disease*" OR "lung disorder*" OR "bronchial disease*" OR "bronchial disorder*" OR "respiratory disease*" OR "respiratory disorder*" OR "pulmonary disease*" OR "pulmonary disorder*" OR "lung" OR "respiratory" OR "pulmonary" OR "bronchial") AND ("blended intervention" OR "blended interventions" OR "blended care" OR "blended approach" OR "blended approaches" OR "blended learning" OR (("blending" OR "blended") AND (intervention* OR "care" OR "healthcare" OR multidisciplin* OR "learning")))) NOT ti=((Infan* OR Child* OR pediatr* OR paediatr*) NOT (Adult* OR elderl*))NOT ti=("veterinary" OR "rabbit" OR "rabbits" OR "animal" OR "animals" OR "mouse" OR "mice" OR "rodent" OR "rodents" OR "rat" OR "rats" OR "pig" OR "pigs" OR "porcine" OR "horse" OR "horses" OR "equine" OR "cow" OR "cows" OR "bovine" OR "goat" OR "goats" OR "sheep" OR "ovine" OR "canine" OR "dog" OR "dogs" OR "feline" OR "cat" OR "cats"))

**Cochrane**

(("Chronic Obstructive Lung Disease" OR "Asthma" OR "COPD" OR "Chronic Obstructive Lung Diseases" OR "Chronic Obstructive Lung Disease" OR "Chronic Obstructive Pulmonary Diseases" OR "Chronic Obstructive Pulmonary Disease" OR "asthma" OR asthma* OR "Respiratory Tract Disease" OR "lung disease*" OR "lung disorder*" OR "bronchial disease*" OR "bronchial disorder*" OR "respiratory disease*" OR "respiratory disorder*" OR "pulmonary disease*" OR "pulmonary disorder*" OR "lung" OR "respiratory" OR "pulmonary" OR "bronchial") AND ("blended intervention" OR "blended interventions" OR "blended care" OR "blended approach" OR "blended approaches" OR "blended learning" OR (("blending" OR "blended") AND (intervention* OR "care" OR "healthcare" OR multidisciplin* OR "learning")))):ti,ab,kw NOT ((Infan* OR Child* OR pediatr* OR paediatr*) NOT (Adult* OR elderl*)):ti

**Emcare**

(("Chronic Obstructive Lung Disease"/ OR exp "Asthma"/ OR "COPD".mp OR "Chronic Obstructive Lung Diseases".mp OR "Chronic Obstructive Lung Disease".mp OR "Chronic Obstructive Pulmonary Diseases".mp OR "Chronic Obstructive Pulmonary Disease".mp OR "asthma".mp OR asthma*.mp OR exp "Respiratory Tract Disease"/ OR "lung disease*".mp OR "lung disorder*".mp OR "bronchial disease*".mp OR "bronchial disorder*".mp OR "respiratory disease*".mp OR "respiratory disorder*".mp OR "pulmonary disease*".mp OR "pulmonary disorder*".mp OR "lung".ti OR "respiratory".ti OR "pulmonary".ti OR "bronchial".ti) AND ("blended intervention".mp OR "blended interventions".mp OR "blended care".mp OR "blended approach".mp OR "blended approaches".mp OR "blended learning".mp OR (("blending".mp OR "blended".mp) AND ("intervention".mp OR "interventions".mp OR "care".ti,ab OR "healthcare".ti,ab OR multidisciplin*.mp OR "learning".mp))) NOT ((exp "Infant"/ OR exp "Child"/) NOT exp "Adult"/)) NOT (conference review or conference abstract).
