# Supplementary material for: Blended Self-Management Interventions to Reduce Disease Burden in Patients With Chronic Obstructive Pulmonary Disease and Asthma: Systematic Review and Meta-analysis
Source: J Med Internet Res. 2021 Mar 31;23(3):e24602. doi: 10.2196/24602 (PMC8047793; doi:10.2196/24602)
Supplement: Multimedia Appendix 2 [file jmir_v23i3e24602_app2.docx]

**Multimedia Appendix 2: Grading of Recommendations, Assessment, Development and Evaluation evidence tables.**

| Outcome indicator | Study | Quality of evidence |
| --- | --- | --- |
|  | | |
| **Health-related effectiveness outcome indicators in COPD (systematic review part)** | | |
| **Mortality** | | High |
|  | Bentley et al. (2014) |  |
|  | Casas et al. (2006) |  |
|  | Sorknaes et al. (2013) |  |
| **Exacerbation frequency** | | Very low |
|  | Jehn et al. (2013) |  |
|  | Koff et al. (2009) |  |
| **BMI** | | Very low |
|  | Garcia et al. (2007) |  |
| **Health-related effectiveness outcome indicators in COPD (meta-analysis part)** | | |
| **Exercise capacity** | | High |
|  | Jehn et al. (2013) |  |
|  | Nguyen et al. (2008) |  |
|  | Wang et al. (2017) |  |
| **Dyspnea** | | Low |
|  | Chau et al. (2012) |  |
|  | Garcia et al. (2007) |  |
|  | Nguyen et al. (2008) |  |
|  | Wang et al. (2017) |  |
| **Lung function** | | Low |
|  | Chau et al. (2012) |  |
|  | Garcia et al. (2007) |  |
|  | Jehn et al. (2013) |  |
|  | Wang et al. (2017) |  |
| **Quality of life** | | Low |
|  | Bentley et al. (2014) |  |
|  | Garcia et al. (2007) |  |
|  | Jehn et al. (2013) |  |
|  | Koff et al. (2009) |  |
|  | Nguyen et al. (2008) |  |
|  | Wang et al. (2017) |  |
|  | Wang et al. (2020) |  |
|  | Xin et al. (2016) |  |
| **Admission** | | Moderate |
|  | Bentley et al. (2014) |  |
|  | Stamenova1 et al. (2020) |  |
|  | Stamenova2 et al. (2020) |  |
|  | Wei et al. (2014) |  |
|  | Xin et al. (2016) |  |
| **Process outcome indicators in COPD (** **systematic review part)** | |  |
| **Visits** | | Low |
|  | Casas et al. (2006) |  |
|  | Chau et al. (2012) |  |
|  | Jehn et al. (2013) |  |
| **Satisfaction** | | Low |
|  | Chau et al. (2012) |  |
| Outcome indicator | Study | Quality of evidence |
|  | Garcia et al. (2007) |  |
|  | Koff et al. (2009) |  |
| **Adherence** | | Very low |
|  | Garcia et al. (2007) |  |
|  | Wei et al. (2014) |  |
|  | Xin et al. (2016) |  |
| **Physical activity** |  | Low |
|  | Garcia et al. (2007) |  |
|  | Nguyen et al. (2008) |  |
| **Self-management** | | Very low |
|  | Nguyen et al. (2008) |  |
|  | Wang et al. (2020) |  |
| **Smoking** | | Very low |
|  | Cameron et al. (2016) |  |
|  | Garcia et al. (2007) |  |
| **Costs** | | Very low |
|  | Bentley et al. (2014) |  |
|  | Koff et al. (2009) |  |
| **Psychosocial** | | Very low |
|  | Cameron et al. (2016) |  |
| **Symptom management** | | Very low |
|  | Cameron et al. (2016) |  |
| **Nutrition** | | Very low |
|  | Cameron et al. (2016) |  |
| **Alcohol** | | Very low |
|  | Cameron et al. (2016) |  |
| **Health-related effectiveness outcome indicators in**  **asthma (systematic review part)** | | |
| **Admission** | | Very low |
|  | Ostojic et al. (2005) |  |
| **BMI** | | Very low |
|  | Türk et al. (2020) |  |
| **Exacerbation frequency** | | Very low |
|  | Ver der Meer et al. (2009) |  |
| **Exercise capacity** | | Very low |
|  | Türk et al. (2020) |  |
| **Health-related effectiveness outcome indicators in**  **asthma (** **meta-analysis part)** | | |
| **Asthma control** | | Moderate |
|  | Cao et al. (2018) |  |
|  | Ver der Meer et al. (2009) |  |
|  | Ver Gaalen et al. 2013 |  |
| **Lung function** | | Moderate |
|  | Cao et al. (2018) |  |
|  | Ostojic et al. (2005) |  |
|  | Türk et al. (2020) |  |
|  | Ver der Meer et al. 2009 |  |
| **Quality of life** | | Moderate |
|  | Cao et al. (2018) |  |
| Outcome indicator | Study | Quality of evidence |
|  | Ver der Meer et al. (2009) |  |
|  | Ver Gaalen et al. (2013) |  |
| Outcome indicator Study | | Quality of evidence |
| **Asthma knowledge** | | Very low |
|  | Meer et al. (2009) |  |
|  | Kohler et al (2020) |  |
| **Visits** | | Very low |
|  | Meer et al. (2009) |  |
|  | Ostojic et al. (2005) |  |
| **Adherence** | | Very low |
|  | Ostojic et al. (2005) |  |
|  | Meer et al. (2009) |  |
