# Supplementary material for: Blended Self-Management Interventions to Reduce Disease Burden in Patients With Chronic Obstructive Pulmonary Disease and Asthma: Systematic Review and Meta-analysis
Source: J Med Internet Res. 2021 Mar 31;23(3):e24602. doi: 10.2196/24602 (PMC8047793; doi:10.2196/24602)
Supplement: Multimedia Appendix 3 [file jmir_v23i3e24602_app3.docx]

**Multimedia Appendix 3: Behaviour change techniques in the blended self-management interventions.**

| Study | | General information | Goal setting | Action planning | Problem-solving/ barrier | Prompt review of  behavioural goals | Prompt self-  monitoring/tracking | Social support | Emotional  control training | Motivational approach | Provide feedback on performance |  |
| --- | --- | --- | --- | --- | --- | --- | --- | --- | --- | --- | --- | --- |
|  | | | | | | | | | | | |  |
| **COPD** | | | | | | | | | | | |  |
|  | Bentley et al.  (2014) | ✓ |  |  | ✓ |  | ✓ |  |  |  | ✓ | |
|  | Cameron et al.  (2016) | ✓ | ✓ | ✓ | ✓ | ✓ | ✓ |  | ✓ | ✓ | ✓ | |
|  | Casas et al.  (2006) | ✓ | ✓ | ✓ | ✓ | ✓ | ✓ | ✓ |  | ✓ | ✓ | |
|  | Chau et al.  (2012) | ✓ |  |  | ✓ |  | ✓ |  |  |  | ✓ | |
|  | Garcia et al.  (2007) | ✓ | ✓ | ✓ | ✓ | ✓ | ✓ | ✓ |  | ✓ | ✓ | |
|  | Haesum et al.  (2017) | ✓ |  |  | ✓ | ✓ | ✓ |  |  |  | ✓ | |
|  | Jehn et al.  (2013) | ✓ |  |  | ✓ |  | ✓ |  |  | ✓ | ✓ | |
|  | Koff et al.  (2009) | ✓ |  |  | ✓ |  | ✓ |  |  | ✓ | ✓ | |
|  | Nguyen et al. (2008) | ✓ | ✓ | ✓ | ✓ | ✓ | ✓ | ✓ | ✓ | ✓ | ✓ | |
|  | Sorknaes et al.  (2013) | ✓ |  | ✓ | ✓ |  | ✓ |  |  | ✓ | ✓ | |
|  | Stamenova et al.(2020) | ✓ | ✓ | ✓ | ✓ | ✓ | ✓ |  |  |  | ✓ | |
|  | Wang et al.  (2017) | ✓ | ✓ | ✓ | ✓ | ✓ | ✓ |  |  | ✓ | ✓ | |
|  | Wang et al.  (2020) | ✓ |  |  | ✓ |  | ✓ | ✓ |  | ✓ | ✓ | |
|  | Wei et al.  (2014) | ✓ |  | ✓ | ✓ |  | ✓ |  |  |  | ✓ | |
|  | Xin et al.  (2016) | ✓ |  | ✓ | ✓ |  | ✓ |  |  |  | ✓ | |
| **Asthma** | | | | | | | | | | | |  |
|  | Barbanel et al.  (2003) | ✓ |  | ✓ | ✓ |  | ✓ |  |  | ✓ |  |  |
|  | Cao et al.  (2018) | ✓ |  |  | ✓ |  | ✓ |  |  |  | ✓ |  |
|  | Gaalen et al.  (2013) | ✓ | ✓ | ✓ | ✓ | ✓ | ✓ | ✓ |  | ✓ | ✓ |  |
|  | Meer et al.  (2009) | ✓ | ✓ | ✓ | ✓ | ✓ | ✓ |  |  | ✓ | ✓ |  |
|  | Kohler et al. (2020) | ✓ |  |  | ✓ |  | ✓ |  |  |  | ✓ |  |
|  | Ostojic et al.  (2005) | ✓ |  |  | ✓ |  | ✓ |  |  |  | ✓ |  |
|  | Türk et al.  (2020) | ✓ | ✓ | ✓ | ✓ | ✓ | ✓ | ✓ | ✓ | ✓ | ✓ |  |
